# Targeting the cell cycle in head and neck cancer by Chk1 inhibition: a novel concept of bimodal cell death

**DOI:** 10.1038/s41389-019-0147-x

**Published:** 2019-06-17

**Authors:** Anne M. van Harten, Marijke Buijze, Richard van der Mast, Martin A. Rooimans, Sanne R. Martens-de Kemp, Costa Bachas, Arjen Brink, Marijke Stigter-van Walsum, Rob M. F. Wolthuis, Ruud H. Brakenhoff

**Affiliations:** 1 Amsterdam UMC, Vrije Universiteit Amsterdam, Otolaryngology/Head and Neck Surgery, Section Tumor Biology, Cancer Center Amsterdam, Amsterdam, The Netherlands; 2 Amsterdam UMC, Vrije Universiteit Amsterdam, Clinical Genetics, Section Oncogenetics, Cancer Center Amsterdam, Amsterdam, The Netherlands

**Keywords:** Head and neck cancer, Apoptosis, Target validation, DNA damage response

## Abstract

Head and neck squamous cell carcinomas (HNSCCs) coincide with poor survival rates. The lack of driver oncogenes complicates the development of targeted treatments for HNSCC. Here, we follow-up on two previous genome-wide RNA and microRNA interference screens in HNSCC to cross-examine tumor-specific lethality by targeting *ATM*, *ATR*, *CHEK1*, or *CHEK2*. Our results uncover *CHEK1* as the most promising target for HNSCC. *CHEK1* expression is essential across a panel of HNSCC cell lines but redundant for growth and survival of untransformed oral keratinocytes and fibroblasts. LY2603618 (Rabusertib), which specifically targets Chk1 kinase, kills HNSCC cells effectively and specifically. Our findings show that HNSCC cells depend on Chk1-mediated signaling to progress through S-phase successfully. Chk1 inhibition coincides with stalled DNA replication, replication fork collapses, and accumulation of DNA damage. We further show that Chk1 inhibition leads to bimodal HNSCC cell killing. In the most sensitive cell lines, apoptosis is induced in S-phase, whereas more resistant cell lines manage to bypass replication-associated apoptosis, but accumulate chromosomal breaks that become lethal in subsequent mitosis. Interestingly, CDK1 expression correlates with treatment outcome. Moreover, sensitivity to Chk1 inhibition requires functional CDK1 and CDK4/6 to drive cell cycle progression, arguing against combining Chk1 inhibitors with CDK inhibitors. In contrast, Wee1 inhibitor Adavosertib progresses the cell cycle and thereby increases lethality to Chk1 inhibition in HNSCC cell lines. We conclude that Chk1 has become a key molecule in HNSCC cell cycle regulation and a very promising therapeutic target. Chk1 inhibition leads to S-phase apoptosis or death in mitosis. We provide a potential efficacy biomarker and combination therapy to follow-up in clinical setting.

## Introduction

Head and neck squamous cell carcinoma (HNSCC) develops in the mucosal lining of the upper aero-digestive tract and comprises ~700,000 (5%) of all newly diagnosed cancer cases worldwide^1^. Smoking, alcohol consumption, and infection with high-risk human papillomavirus (HPV) are known risk factors for HNSCC^2^, and despite invasive treatment protocols, the 5-years survival rate of HNSCC patients remain around 60%^2,3^.

Standardized treatment protocols comprise surgical resection of the tumor, radiotherapy, and platinum-based concomitant chemoradiation, often in combination, resulting in severe side effects^2^. The only targeted therapy approved for HNSCC is cetuximab, a chimerized monoclonal antibody against EGFR^4^. However, response predicting biomarkers are not known^5^. New therapies are urgently awaited to reduce toxicities, improve survival rates, and quality of life.

Recently, the TCGA published a comprehensive molecular landscape of somatic mutations in HNSCC^6^. The lack of oncogenic mutations hampers the identification of therapeutic targets, but the large number of mutations in cell cycle related tumor suppressor genes pinpoints the altered cell cycle as a promising HNSCC druggable target (reviewed in Leemans et al.)^7^. First, *TP53* is altered in the large majority of HNSCC, due to mutations or inactivation by the HPV E6 oncoprotein^6^. Additionally, *CDKN2A*/p16 function is lost and Cyclin D1 often overexpressed, which together result in a dysfunctional G1/S-checkpoint and a compromised G2/M-checkpoint^2,6^. Loss of G1/S regulation causes unscheduled S-phase entry, induces replication stress that often results in DNA damage, and causes the cell cycle control to predominantly rely on S-phase and G2/M regulation.

When DNA damage occurs in normal cells, repair is initiated by canonical ATM/ATR pathway activation. When double-stranded DNA breaks (DSBs) are detected, ATM is activated by the Mre11-Rad50-Nbs1 (MRN) complex, and subsequently Chk2 is activated. ATR and Chk1 activation is induced by stalled replication forks and single-stranded DNA^8–11^. In both scenarios, cell cycle arrest is initiated followed by activation of DNA repair signaling cascades such as non-homologous end joining (NHEJ) and homologous recombination (HR)^8–11^. Furthermore, ATR and Chk1 play an important role during DNA replication in S-phase by stabilization of the replication forks^8,12–14^. Chk1 regulates the firing of replication origins during S-phase, but seems to be more broadly involved^8,12–14^. The ATM and ATR DNA damage response pathways are not completely redundant, but overlap in downstream regulators might compensate the loss of one pathway^9^. Whether these systems work accordingly in tumor cells with an abrogated cell cycle is unclear.

Targeting the DNA damage response in relation to the rewired cell cycle in cancer cells is a promising approach for therapy^11^. Abrogated cell cycle control is a typical hallmark for most cancer cells, particularly for HNSCC, and several lines of evidence suggest a synthetic lethality between *TP53* mutations and Chk1 inhibition in triple-negative breast cancer^15–17^.

**Fig. 1:**
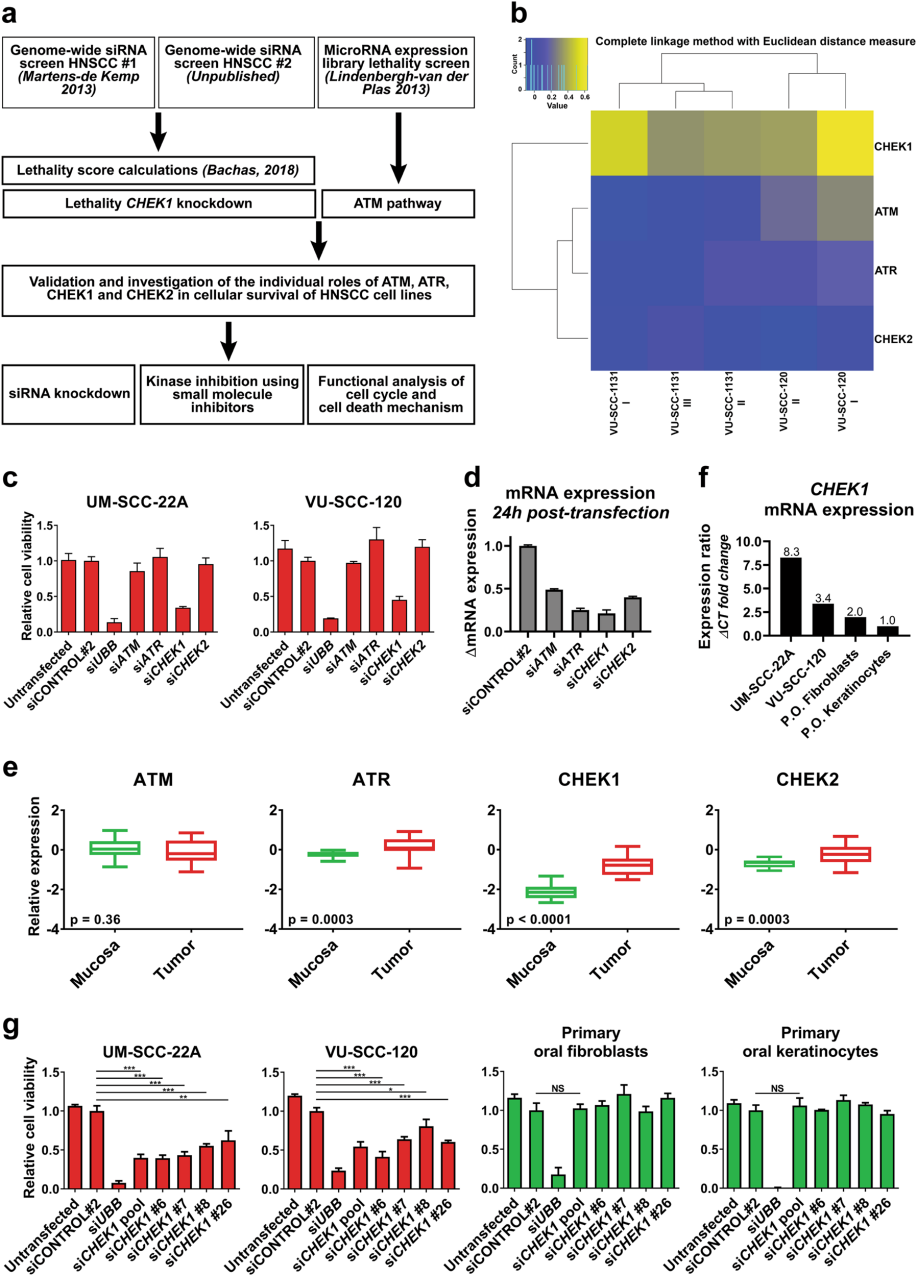
RNA interference of *CHEK1* decreases cell viability in HNSCC cell lines, but not in primary oral keratinocytes and fibroblasts. **a** Overview of the workflow presented in this manuscript. **b** Heatmap representing the lethality score^20^ of *ATM, ATR, CHEK1, CHEK2* from the individual replicates of the genome-wide siRNA screen, independently performed in HNSCC cell lines VU-SCC-1131 and VU-SCC-120. Blue represents no effect on viability, yellow represents the decrease in viability. FDR corrected *p*-value cutoff: 0.005. **c** Transfections with SMARTpools containing four pooled siRNA sequences targeting either *ATM, ATR, CHEK1*, or *CHEK2* demonstrated that only si*CHEK1* decreased cell viability for ≥50% (UM-SCC-22A and VU-SCC-120 relative viability 0.34 and 0.45, respectively). Knockdown of si*ATM*, si*ATR*, and si*CHEK2* did not reduce cell viability in tested cell lines (relative average viability UM-SCC-22A, respectively, 0.86, 1.06, 0.96; for VU-SCC-120, respectively, 0.97, 1.30, 1.20). siCONTROL#2 was transfected as negative control, si*UBB* targeting Ubiquitin B as positive control. **d** Knockdown of *ATM, ATR, CHEK1*, and *CHEK2* was analyzed 24 h post transfection in VU-SCC-120 by RT-qPCR. Expression was normalized for *GUSB* and relative to the siCONTROL#2. Values were 0.49, 0.25, 0.21, and 0.40, respectively. **e** Microarray gene expression data of 22 tumors (red boxplots) with paired normal mucosa (green boxplots) revealed a significant increase of *ATR*, *CHEK1*, and *CHEK2* expression in tumors at the RNA level, but not for *ATM*. Data are represented as boxplots with on the *y*-axis the relative expression against the reference RNA^34^. The horizontal line represents the median value. **f** Basal *CHEK1* mRNA expression levels were compared between primary oral keratinocytes and fibroblasts and tumor cell lines UM-SCC-22A and VU-SCC-120. A relative fold change expression ratio was calculated towards the basal *CHEK1* expression in the keratinocytes. Fibroblasts expressed a two-fold increase in *CHEK1*, VU-SCC-120 a 3.4-fold increase, and UM-SCC-22A an 8.3-fold increased expression. **g** Deconvolution of the four individual SMARTpool *CHEK1* siRNAs on two HNSCC cell lines (red bars) and primary oral keratinocytes and fibroblasts (both represented in green). A significant decrease in cell viability was observed in the HNSCC cell lines (two-sided *t*-test *p*-values versus siCONTROL#2 viability for UM-SCC-22A: si*CHEK1* pool: 0.0002, si*CHEK1* #6: 0.0002, si*CHEK1* #7: 0.0003, si*CHEK1* #8: 0.0004, si*CHEK1* #26: 0.0092. For VU-SCC-120: si*CHEK1* pool: 0.0005, si*CHEK1* #6: 0.0002, si*CHEK1* #7: 0.0003, si*CHEK1* #8: 0.0276, si*CHEK1* #26: 0.0002.). No significant reduction in viability was obtained upon *CHEK1* knockdown in the primary mucosal cells, while the positive control si*UBB* was lethal in all cells tested

In functional genomic screens, *ATM* and *CHEK1* emerged as essential genes in HNSCC^18,19^. In this study, we cross-validated *ATM, ATR, CHEK1*, and *CHEK2* as potential targets for therapy, and their role in cell cycle regulation in normal and malignant squamous cells (Fig. 1a).

## Results

### Specifically Chk1 abrogation impacts HNSCC cells

First, we reanalyzed two independent genome-wide screens for the effects of *ATM*, *ATR*, *CHEK1*, and *CHEK2* siRNAs by a novel lethality score calculation^20^. This revealed that particularly *CHEK1* knockdown significantly decreased cell viability in HNSCC cell lines (Fig. 1b and S1a). Follow-up experiments confirmed that *CHEK1* knockdown causes a significant reduction of cell viability, whereas knockdown of *ATM, ATR*, or *CHEK2* had only limited effects in concordance with the screening data (compare Fig. 1c with 1b). Knockdown of Ubiquitin B (*UBB)* was used as positive transfection control, siCONTROL#2 as negative control to observe transfection-induced toxicity. Analysis of mRNA levels confirmed that knockdown was 50% or more for all genes (Fig. 1d).

Next, we analyzed the expression levels of these same genes in array data of 22 paired HPV-negative oral cancers and oral mucosa to investigate changed expression in malignant cells, and showed a highly significant 2.7-fold upregulation of *CHEK1* mRNA in cancers as compared to oral mucosa. *ATR* was 1.5-fold increased, and *CHEK2* 1.8-fold increased. Expression levels of *ATM* were not significantly altered (Fig. 1e).

These experiments strongly pinpointed *CHEK1* as most interesting target in HNSCC. *CHEK1* mRNA expression is 8.3- and 3.4-fold increased in cell lines UM-SCC-22A and VU-SCC-120, respectively, compared to primary keratinocytes and in line with the patient expression data (compare Fig. 1f with 1e). Deconvolution of the *CHEK1* siRNA SMARTpool in an extended panel of HNSCC lines, resulted in significant reduction of cell viability for each *CHEK1* siRNA, confirmed by mRNA knockdown (Fig. 1g and S1b, c). Importantly, viability of primary oral fibroblasts and keratinocytes was not significantly affected by *CHEK1* knockdown. This observation does not relate to population doubling times of the primary cells, as proliferation rates of all tested cells are within a similar range, between 20 and 27 h depending on the donor^21,22^.

### Tumor-specific cytotoxicity by small molecule inhibition of Chk1

To further investigate the potential druggability of these genes in HNSCC, we tested several kinase inhibitors. Small molecule inhibitors of ATM (KU-60019, Wortmannin) (Fig. S2a, b) and ATR (ETP-46464 (a dual ATR and mTOR inhibitor), VE-821) (Fig. S2c, d) only reduced cell viability at high drug concentrations. More importantly, there was no therapeutic window obtained between non-transformed mucosa-derived keratinocytes and fibroblasts and HNSCC (Fig. S2a, d). This most likely relates to lack of specificity of the small molecule inhibitors.

**Fig. 2:**
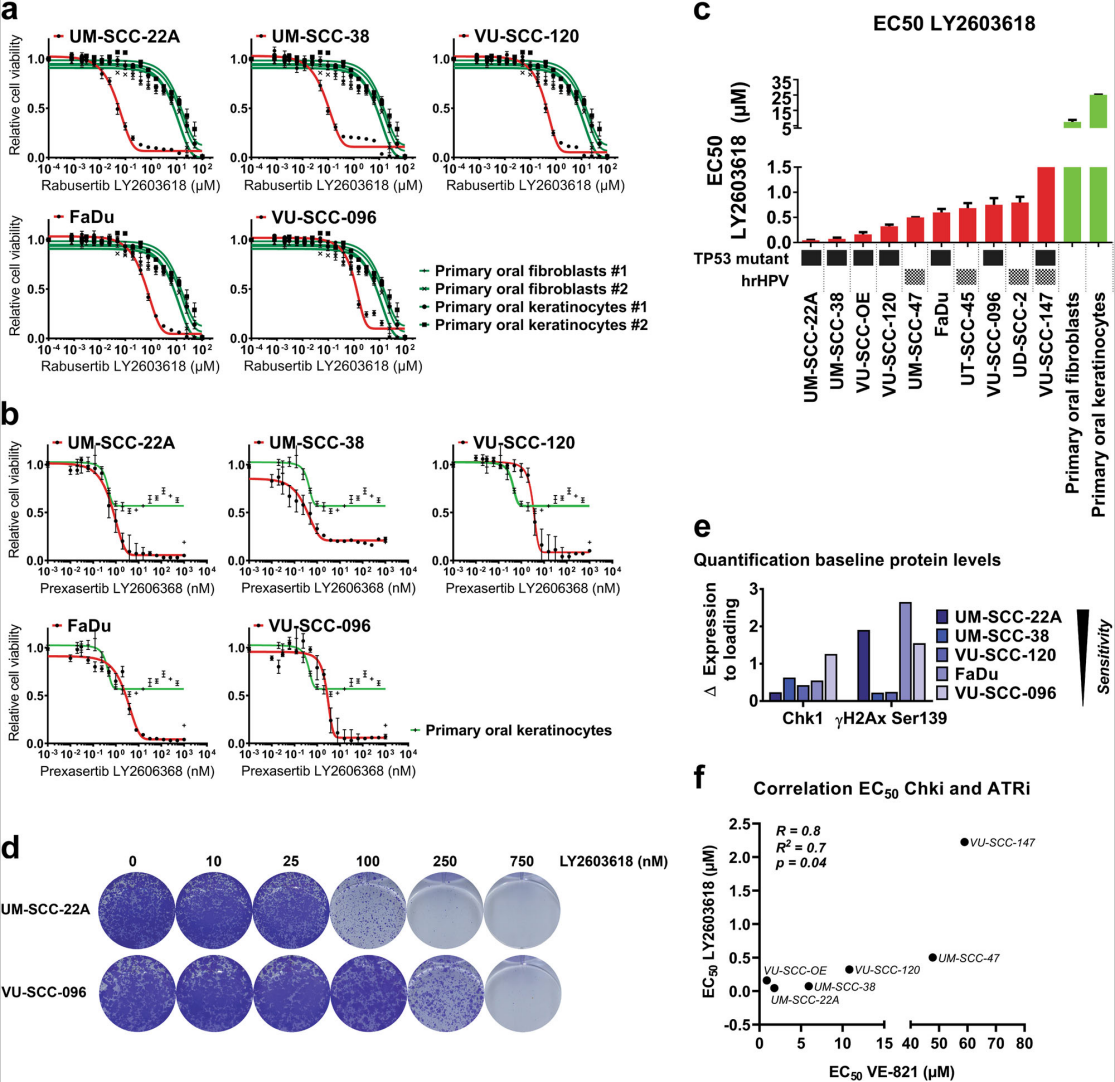
Tumor-specific cytotoxicity through small molecule inhibition of Chk1 in vitro. **a** Dose–response curves shows relative cell viability of HNSCC cell lines (red line) and untransformed primary oral fibroblasts and primary oral keratinocytes (two individual donors each, green lines) for the Chk1 inhibitor LY2603618/Rabusertib (72 h exposure). Experiments were performed three times in triplicate and the averaged value is indicated. Note the therapeutic window between tumor and primary cells, indicating tumor-specific cytotoxicity of Chk1 inhibition. **b** Treatment with LY2606368/Prexasertib, a dual Chk1/Chk2 inhibitor, resulted in cytotoxic effects on HNSCC cells (in red) and primary oral keratinocytes (in green), but no therapeutic window was found. The increased viability of the keratinocytes at higher concentrations suggests an off-target effect. **c** Half maximal effective concentrations (EC_50_) of LY2603618/Rabusertib represented per tested HNSCC cell lines (red bars) and primary mucosal cell type (green bars). *TP53* mutational status, and presence of hrHPV are depicted below and in Table 1. **d** Long-term exposure (10 days) of LY2603618/Rabusertib indicated an intrinsic difference in sensitivity for the most sensitive (UM-SCC-22A) and moderately sensitive (VU-SCC-096) HPV-negative HNSCC cell lines. After drug treatment, cells were fixed and stained with crystal violet in situ. **e**. Quantification of protein levels (Fig. S3a) did not reveal a correlation between either Chk1 expression levels or basal DNA damage levels measured by γH2Ax Ser139 (Fig. S3b, c). Protein levels were normalized by the loading control HSP90α/ß. Cell lines are ordered to their sensitivity to Chk1 inhibition (left to right, most to less sensitive). **f** EC_50_ values of four HPV-negative and two HPV-positive HNSCC cell lines were determined for Chk1 inhibitor LY2603618/Rabusertib and ATR inhibitor VE-821. Pearson correlation showed a significant correlation between responses to ATR inhibition and Chk1 inhibition, which was expected since Chk1 is a direct substrate of ATR. However, no therapeutic window was found for ATR inhibition with primary cells (Fig. S2c, d), which may relate either to the specificity of the inhibitors, or the apparent novel role of Chk1 in malignant cells

In parallel, four clinically relevant Chk1 inhibitors were tested: MK-8776 (SCH 900776), PF-477736, LY2603618/Rabusertib, and LY2606368/Prexasertib (Fig. 2a, b and S2e–g). It was recently established by Klaeger et al.^23^ that LY2603618/Rabusertib is the most specific Chk1 inhibitor^24^, which is in line with the dose–response curves (Fig. 2a–c and S2g). LY2606368/Prexasertib is a presumed Chk1 inhibitor, but targets at least both Chk1 and Chk2^25^. LY2606368/Prexasertib had no therapeutic window between primary cells and HNSCC cell lines, which might relate to dual Chk1/Chk2 inhibition or off-target effects as seen with other Chk1 inhibitors (Fig. 2b and S2e–g).

As LY2603618/Rabusertib was the most selective Chk1 kinase inhibitor in this comparison^23–25^, half maximal effective concentration (EC_50_) values were determined on an extended cell line panel (Fig. 2c, S2g, and Table 1). All HPV-negative lines exhibit both a *TP53* mutation and loss of at least one *CDKN2A* locus (Table 1). Three HPV-negative HNSCC lines (UM-SCC-22A, UM-SCC-38 and VU-SCC-OE) were very sensitive to Chk1 inhibition with EC_50_ < 200 nM after 72 h treatment. The other HNSCC lines tested were moderately sensitive (EC_50_ 200–800 nM), and one HPV-positive line VU-SCC-147 was resistant (EC_50_ 2.3 ± 0.7 µM). The primary oral fibroblasts and keratinocytes had an EC_50_ > 2.5 µM, harmonious with viability after *CHEK1* knockdown (Fig. 1f, S1b, S2g, and Table 1).

**Table 1:** *TP53* mutation status, *CDKN2A*/p16 loss, and EC_50_ values ± standard error of the mean (SEM) of LY2603618/Rabusertib treatment per cell line

Cell line	*TP53* mutation^a^	*CDKN2A* (p16) loss	EC_50_ (µM) LY2603618/ Rabusertib
UM-SCC-22A	g. 13419A>G g. 14754+1G>T	Double loss	0.045 ± *0.008*
UM-SCC-38	g. 13075G>T	Loss	0.073 ± *0.027*
VU-SCC-OE	g. 11727_14754del	Double loss	0.160 ± *0.047*
VU-SCC-120	g. 13160/13161 GC>TT g. 13206G>A	Double loss	0.322 ± *0.035*
UM-SCC-47	–	–	0.502 ± *0.001*
FaDu	g. 14070G>T	Loss	0.598 ± *0.071*
UT-SCC-45	–	–	0.686 ± *0.099*
VU-SCC-096	g. 13338A>T	Loss	0.751 ± *0.133*
UD-SCC-2	–	Loss	0.800 ± *0.112*
VU-SCC-147	g. 14097T>G	–	2.225 ± *0.745*

^a^IARC TP53 Database Download TP53 Somatic R12 Genbank: X54156, published in Martens-de Kemp et al*.*^26^

A 16-fold difference in EC_50_ was found between HPV-negative HNSCC cell lines UM-SCC-22A and VU-SCC-096. The moderate sensitivity of VU-SCC-096 remained after drug exposure for 10 days (Fig. 2d). These different drug responses are not explained by population doubling rates, which is 22 h for both cell lines^22^. Other explanations such as Chk1 protein expression levels or the presence of intrinsic DNA damage (γH2Ax Ser139) did not correlate significantly with sensitivity (Fig. 2e and S3a–c). Furthermore, neither HPV-status, nor *TP53* mutation status^26^, nor *CDKN2A*/p16 expression levels or losses explained the differences in HNSCC sensitivity to Chk1 inhibition (Table 1 and Fig. S3d, e). In conclusion, our data show that specific Chk1 inhibition is preferred over dual Chk1/2 inhibition, albeit sensitivities to Chk1 inhibition differ between cell lines.

Since Chk1 is a direct substitute of ATR, we investigated the correlation between the sensitivities of the most specific inhibitors tested, ATR inhibitor VE-821 and Chk1 inhibitor LY2603618/Rabusertib (Fig. 2f). In a panel of 6 HNSCC lines, the sensitivities correlated significantly (*R*^2^ = 0.7, *p* = 0.04). However, ATR inhibition did not result in a therapeutic window between HNSCC and primary cells, as established with Chk1 inhibition, which may relate to the specificity of the inhibitors or a novel role of Chk1 in HNSCC.

### HNSCC cells arrest in S-phase upon Chk1 inhibition

**Fig. 3:**
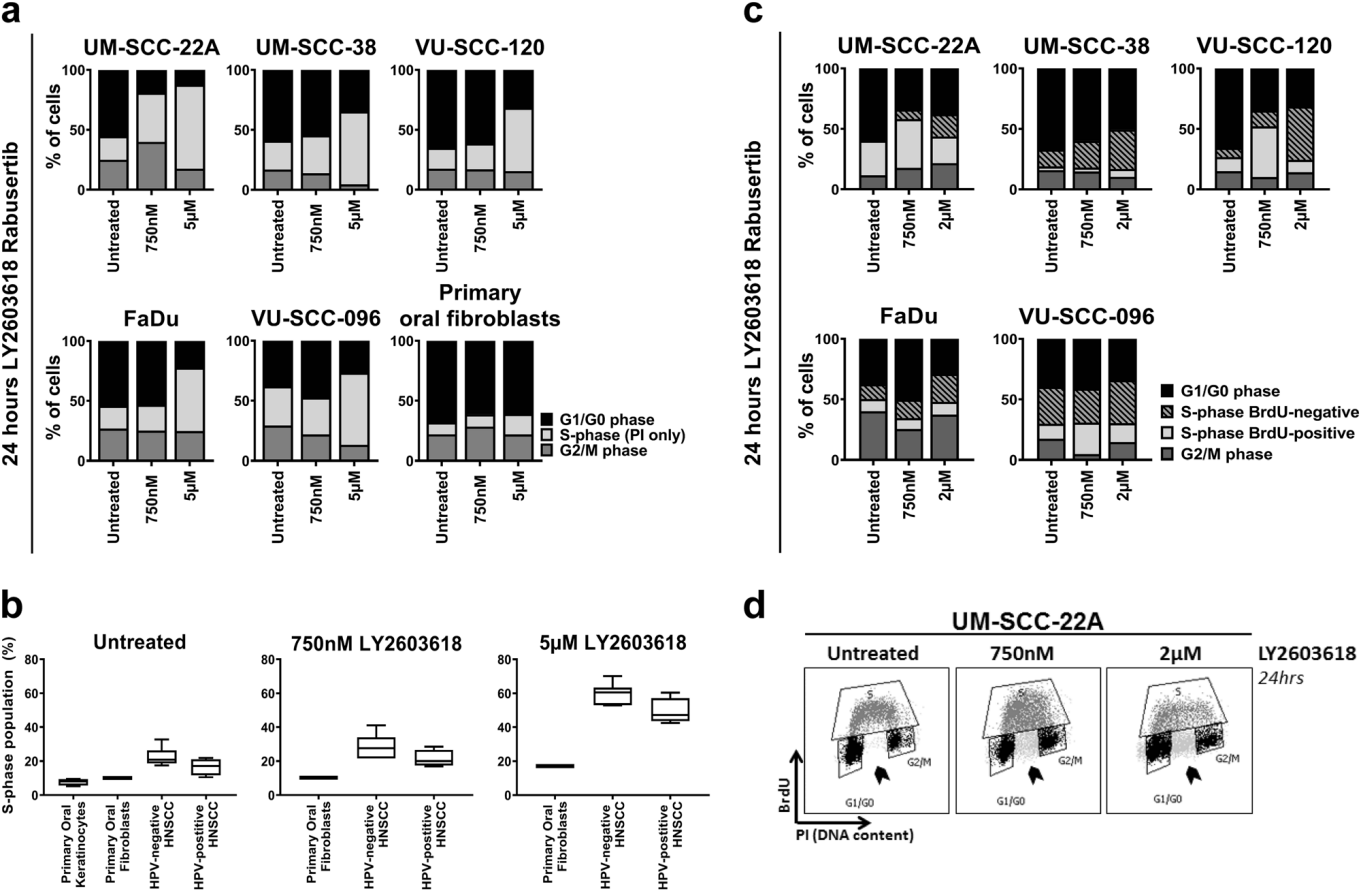
Chk1 inhibition results in an increasing S-phase population due to replication problems. **a** A general cell cycle distribution was obtained after 24 h of LY2603618/Rabusertib treatment of HNSCC cell lines and primary oral fibroblasts. After RNAse incubation, the DNA content was stained with propidium iodide (PI). For all cell lines, an increased S-phase population was observed. Relative DNA content is depicted. An extended panel of HNSCC cell cycle profiles upon LY2603618/Rabusertib treatment is depicted in Fig. S3f. **b** Mean S-phase population determined by PI FACS analysis of primary oral fibroblasts, HPV-negative HNSCC cell lines, and HPV-positive HNSCC cell lines. S-phase population of untreated primary oral keratinocytes were obtained from previously published data^21^. Untreated and LY2603618/Rabusertib treated populations are shown. All HNSCC cell lines, except UM-SCC-47, contained a higher S-phase population compared with the primary mucosal cells. Upon treatment, the S-phase population of the HNSCC cells increases drastically, where the primary fibroblast S-phase population remains small (untreated 10%; 750 nM 10.2%; 5 µM 17.1%). Generally, HPV-negative cell lines showed the largest S-phase population, in all conditions tested, suggesting severe replication stress. **c** Cell cycle analysis of DNA replication by BrdU and DNA content by PI. The total S-phase population is represented in two populations; BrdU-positive and BrdU-negative. Striking is the increasing population of non-replicating cells, that did proceed from G1 to S-phase, but were not able to incorporate BrdU during the pulse. These non-replicating cells in untreated cells represent baseline replication stress, which is enhanced by Chk1 inhibition in all cell lines. **d** Representable gating example of FACS analysis. Arrow heads depict the non-replicating cells in S-phase that are negative for BrdU. The lower graphs show the gated event graphs of the G1/G0, S (BrdU-positive cells only) and G2/M populations from upper squatter plots

The different sensitivities to LY2603618/Rabusertib between cell lines warranted further investigation. Chk1 plays an evolutionary conserved role in cell cycle regulation^8,12–14^, therefore, cell cycle distribution was assessed by DNA content analysis (propidium iodide (PI), Fig. 3a and S3g). After 24 h of Chk1 inhibition, all HNSCC cell lines exhibited an increase in DNA content that could relate to either accelerated entry or delayed exit of S-phase. The latter seemed most plausible given the reduced proliferation rates upon Chk1 inhibition and the increased S-phase population appearing 8 h after treatment, which is in line with the average duration of S-phase (Fig. S3h)^27^. This strongly suggests that DNA replication problems occur in early S-phase and subsequently accumulate in the cells. Furthermore, HPV-positive and HPV-negative lines both revealed an increased S-phase population (Fig. 3b).

Next, we investigated the S-phase delay induced by Chk1 inhibition with BrdU incorporation. We observed a large population of non-replicating cells with a DNA content between 2N and 4N that failed to synthesize any DNA during the 15 min BrdU labeling (Fig. 3c, d), suggesting replication stalling and fork collapse (Fig. 3d). This intrinsic DNA replication problem, further aggravated by Chk1 inhibition, was observed in all tested HNSCC cell lines (Fig. 3c). Comparable S-phase problems were obtained with LY2606368/Prexasertib (Fig. S3i).

In these experiments, we noted that all cell lines (except UM-SCC-47) showed an intrinsically increased S-phase fraction, suggesting reduced progression, as compared to primary cells. S-phase populations of HPV-negative HNSCC lines were on average 23% (17–33%), for HPV-positive HNSCC lines 17% (11-22%), while for primary keratinocytes^21^ it was 7.7% (5.2–9.4%) and 10% for primary fibroblasts (Fig. 3a, b and S3g). Only a slight increase in the population of S-phase fibroblasts was observed upon treatment. Hence, the endogenous replication problems of HNSCC cells are tremendously enhanced by Chk1 disruption, likely explaining the efficacy and working mechanism of specific Chk1 targeting.

### Time-lapse microscopy reveals bimodal HNSCC cell killing by Chk1 inhibition

**Fig. 4:**
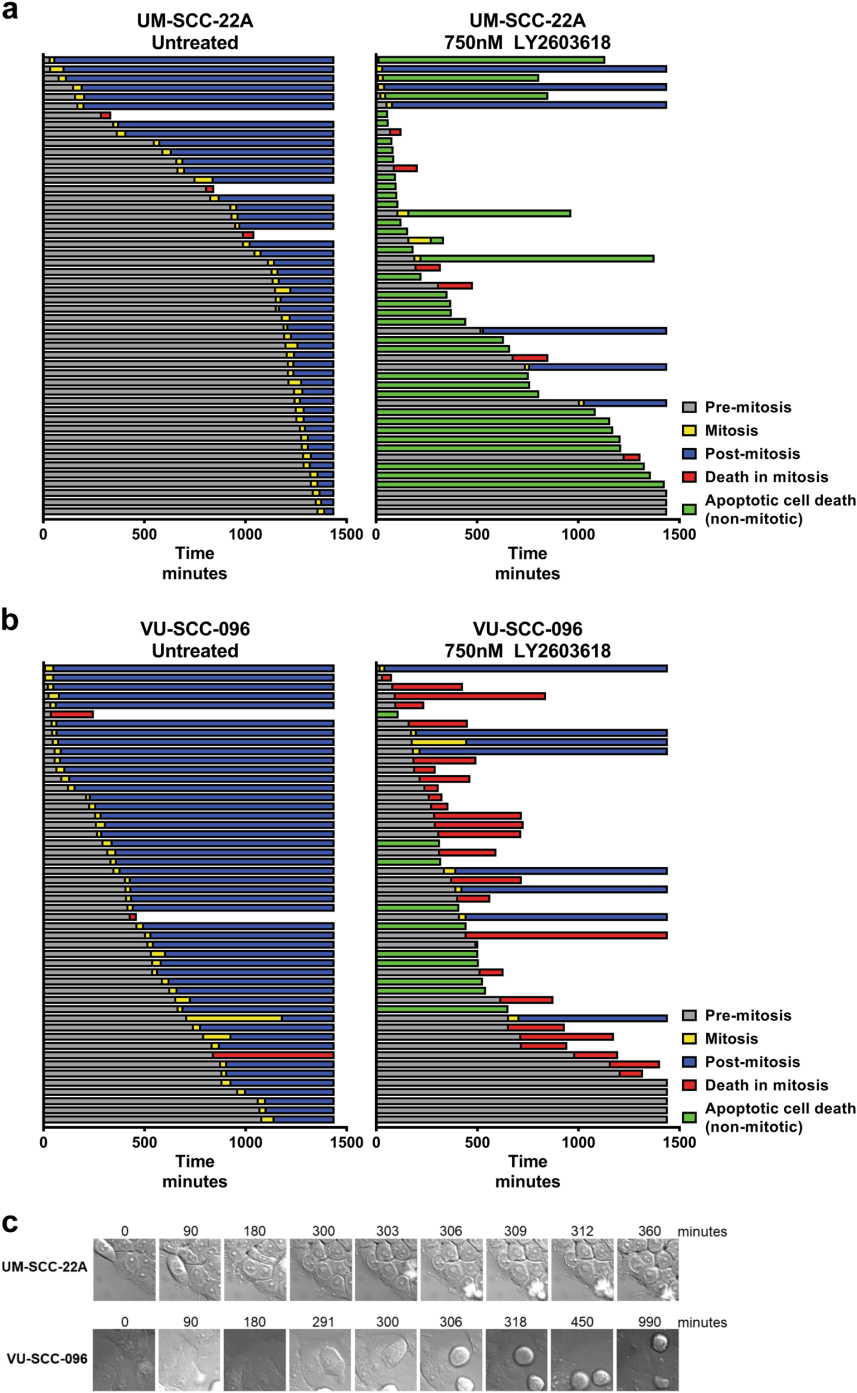
Time-lapse microscopy reveals either an apoptotic non-mitotic cell death or death in mitosis. Using live cell imaging, HNSCC cell lines were followed for 24 h per condition. Each horizontal bar represents a single cell that was tracked over time (in minutes) at the *x*-axis. Pre-mitosis (gray bars) represents the time a cell spent in G1/G0-, S- and G2-phase before mitosis (yellow bars). Post-mitosis (blue bars) is the time a cell spent in G1/G0-, S- and G2-phase after mitosis combined. Bars are ranked to length of the pre-mitotic phase. **a** Mitotic cell death (red bars) was observed in 3 of 50 cells of the sensitive cell line UM-SCC-22A without treatment. However, with LY2603618/Rabusertib treatment, only 6 of 50 cells died by death in mitosis (red bars), whereas 33 of 50 cells underwent non-mitotic cell death characterized by blebbing (green bars). **b** Of the less-sensitive cell line VU-SCC-096, mitotic cell death (red bars) occurred in 3 of 50 cells when left untreated. Treatment with LY2603618/Rabusertib caused death in mitosis in 27 of 50 cells (red bars) and in 10 of 50 non-mitotic cell death (green bars). **c** Representable example of observed cell death mechanisms with live-cell imaging. UM-SCC-22A cells undergo non-mitotic cell death by displaying apoptotic-bodies (blebbing). Within similar timespan, VU-SCC-096 cells enter mitosis that lasted for ~12 h, followed by mitotic cell death

To unravel the working mechanism of Chk1 inhibition, we used time-lapse microscopy to quantitatively investigate the different cell cycle phases. We compared two cell lines with different drug sensitivities (UM-SCC-22A; EC_50_ = 0.045 µM, VU-SCC-096; EC_50_ = 0.75 µM). Cells were filmed during 24 h at three minutes intervals. We analyzed 50 cells per condition (Fig. 4a, b). For both cell lines, three of 50 untreated cells underwent mitotic cell death (left panels). This was not observed when filming untransformed cells (Table S1, data not shown)^28^, again demonstrating intrinsic replication stress in HNSCC cells (Fig. 3).

In untreated cells, mitosis occurred in a normal time frame, ~45 min as previously reported (Fig. S4a, b)^29^. Importantly, after treatment, most of the sensitive UM-SCC-22A cells underwent blebbing and subsequent apoptosis (Fig. 4c, a and Table S1), which occurred before entering mitosis (Fig. 4a, green bars; 33 of 50 UM-SCC-22A cells). Only six of 50 cells filmed, reached mitosis within 3 h after treatment (yellow and red bars). Intriguingly, even when cells managed to enter mitosis, cell death followed during mitosis (Fig. 4a, S4a, and Table S1).

Our FACS analyses revealed that Chk1 inhibition triggers stalled DNA replication. We therefore infer that Chk1 inhibition arrests UM-SCC-22A after which cells become apoptotic in or right after S-phase, caused by replication problems. In contrast, the moderately sensitive VU-SCC-096 cells almost all progressed to mitosis in an apparently normal time frame (Fig. 4b, yellow and red bars), where they stalled for ~4.5 h (Fig. 4b, c, S4b, and Table S1). In total, 27 of 50 VU-SCC-096 cells died in mitosis after this marked delay. Only 10 of 50 cells underwent the S-phase-related apoptosis. These results indicate that specific Chk1 inhibition exerts a dual mode of action in HNSCC cells: either inducing apoptosis as a direct consequence of S-phase replication problems, or mitotic death in case they manage to resist apoptosis and progress through G2/M, which is a common hallmark of cancer^17^.

### Chk1 inhibition activates either caspase 3/7, or induces chromosomal breakage

**Fig. 5:**
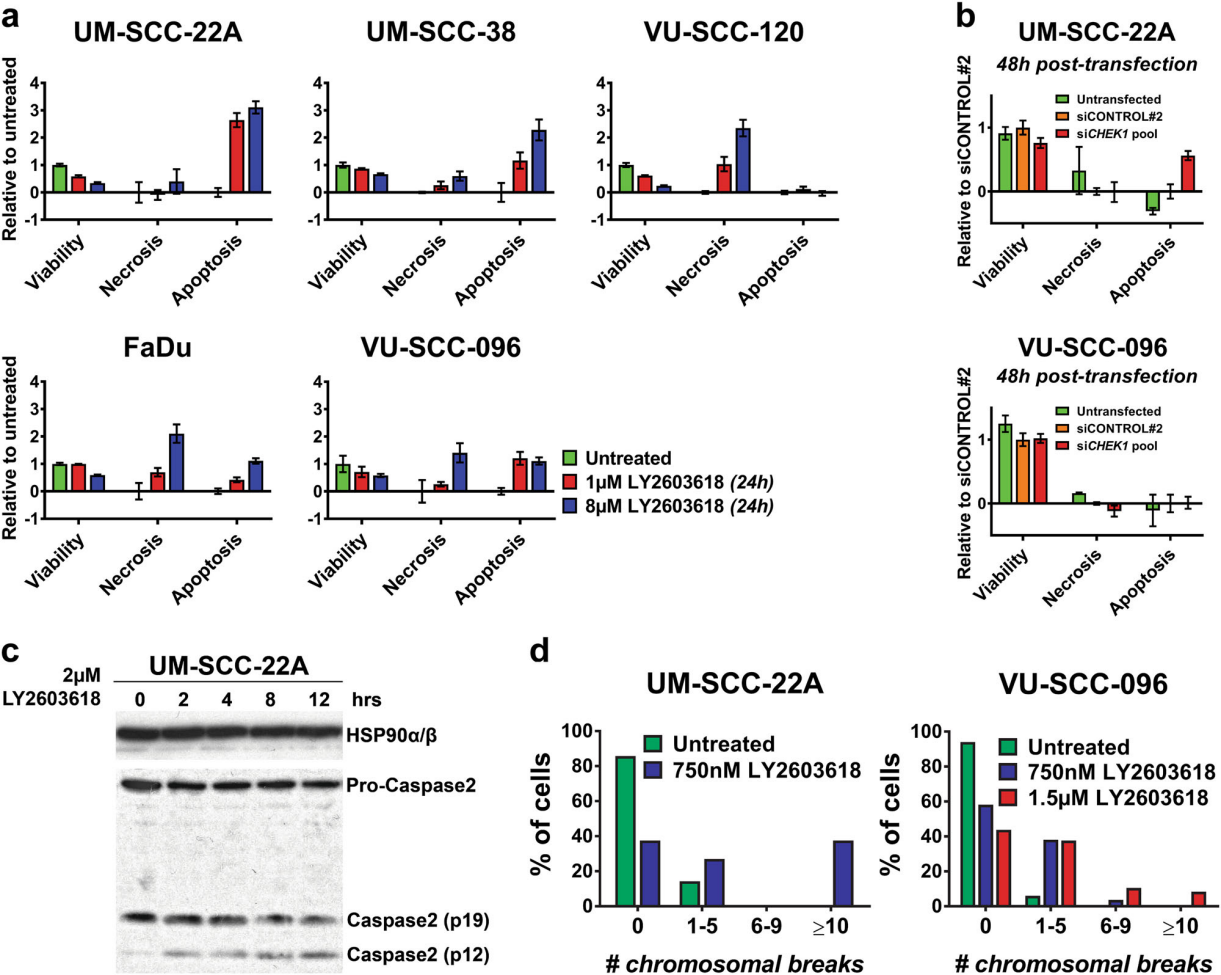
Chk1 inhibition initiates caspase-activation in sensitive cell lines and increased chromosomal breakage in moderately sensitive cells. **a** Sensitive HNSCC cell lines UM-SCC-22A and UM-SCC-38 harbor high levels of caspase 3/7 activity after 24 h of Chk1 inhibition. Induction of necrosis was observed in HNSCC cell lines VU-SCC-120 and FaDu, which is related to death in mitosis, whereas VU-SCC-096 showed both necrosis and some apoptosis. Responses were dose-dependent. **b** After *CHEK1* knockdown (red bars), sensitive cell line UM-SCC-22A expresses increased caspase 3/7 activity 48 h after transfection. This was not observed in less-sensitive cell line VU-SCC-096 in accordance with small molecule inhibition with LY2603618/Rabusertib. Untransfected (green bars) and siCONTROL#2 (orange bars) conditions were included as controls. **c** Caspase 2 is an initiator of apoptotic cell death, and was found to be activated in UM-SCC-22A, likely because of the high levels of DNA damage (Fig. S5a). This cell line also showed the highest caspase 3/7 activity upon Chk1 inhibition and knockdown (Fig. 5a, b and S4c). **d** Metaphase cells exhibited a high number of chromosomal breaks in both sensitive as moderately sensitive cell lines. However, mitotic cells could not be scored for UM-SCC-22A after 1.5 µM LY2303618/Rabusertib treatment for 24 h, indicating pre-mitotic cell death in line with the findings from the live-cell imaging. The number of chromosomal breaks in VU-SCC-096 upon Chk1 inhibition is in line with mitotic cell death observed with live-cell imaging and is considered to be lethal for any cell

To further investigate cell death in a larger panel of cell lines and to exclude dose-dependent cell death, we performed an ApoTox-Glo Triplex assay (Promega) with multiple concentrations of LY2603618/Rabusertib (Fig. 5a and S4c). Sensitive cell lines UM-SCC-22A and UM-SCC-38 both showed a rise in active caspase 3/7, a known marker for apoptosis execution, in relation to an increasing concentration of LY2603618/Rabusertib after 24 h, with a negligible increase of caspase-independent cytotoxicity (Fig. 5a). The moderately drug-sensitive lines VU-SCC-120, FaDu and VU-SCC-096 exhibited an increase in necrotic cells that can be explained by mitotic cell death, and little increase of caspase 3/7 activity. These findings remained consistent in a range of drug concentrations (Fig. S4c), implying that apoptosis is not induced at higher drug concentrations. Caspase 3/7 activity was also induced in UM-SCC-22A 48 h post transfection with si*CHEK1*, but not in VU-SCC-096 (Fig. 5b).

An increase in DNA DSBs is associated with apoptotic cell death via caspase 2 activation^30^. Caspase 2 is an apoptotic initiator, although the exact function and regulation remain unclear. Levels of p-ATM Ser1981 are assumed to play an inducing role via alternative routes^31^, and Western blot analysis indeed demonstrated an increase in p-ATM Ser1981 upon Chk1 inhibition in UM-SCC-22A (Fig. S5a). Also activation of caspase 2 (both p12 and p19) was observed in these cells between 2 h and 12 h of Chk1 inhibition (Fig. 5c). This shows that a pre-apoptotic signaling cascade, possibly associated with DNA damage, is induced.

Subsequently, we investigated DNA damage detection in mitotic cells. Chromosomal breakage analysis of metaphase cells confirmed an increased number of DNA DSBs after 24 h of Chk1 inhibition (Fig. 5d). Only few cells of sensitive line UM-SCC-22A entered metaphase during the course of the assay, due to pre-mitotic cell death. Of these few cells, 64% displayed one or more chromosomal breaks, with 37% of cells containing ≥10 chromosomal breaks (Fig. 5d, left panel). We were not able to score 50 metaphases in UM-SCC-22A at a higher inhibitor concentration. The moderately sensitive line VU-SCC-096 harbored an exceptionally high number of chromosomal breaks after treatment with both 750 nM and 1.5 µM LY2603618/Rabusertib (42% and 56%, respectively). This amount of DNA damage is incompatible with successful anaphase and cytokinesis, causing death in mitosis.

### CDK1 levels are indicative for response

**Fig. 6:**
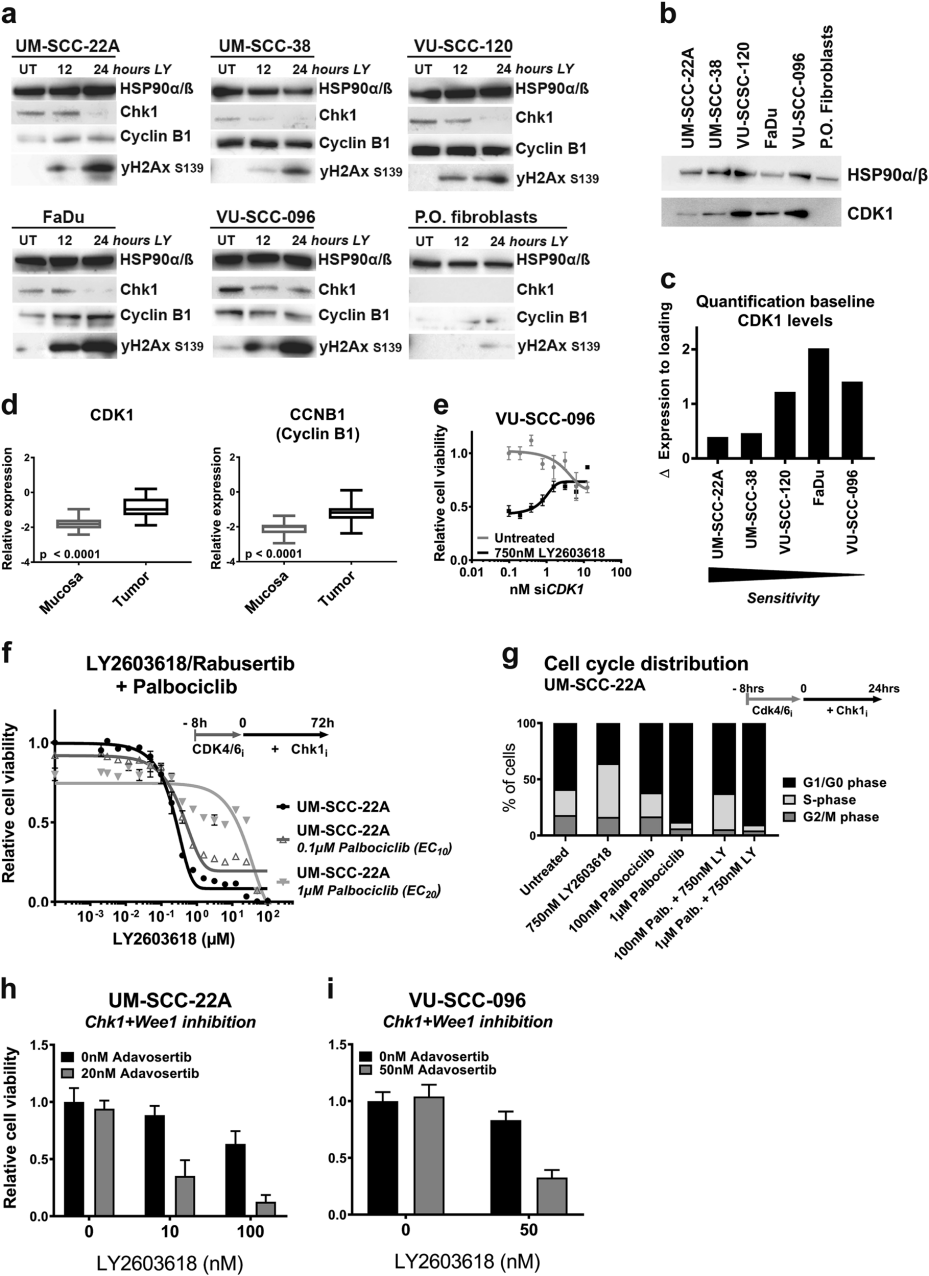
Molecular pathway analysis of cell cycle regulators indicated CDK1 as therapeutic biomarker for Chk1 response. **a** Levels of cell cycle regulating proteins of five HNSCC cell lines and one primary oral fibroblast culture were examined on Western blot. Cells were harvested 12 h and 24 h after treatment with 2 µM LY2603618/Rabusertib. Phosphorylated γH2Ax Ser139 was found in all cell lines after 24 h Chk1 inhibition indicating DNA damage, but was almost absent in primary cells. Cyclin B1 protein levels did not correlate to sensitivity for Chk1 inhibition. **b** Baseline proteins levels of CDK1 are shown for HNSCC cell lines UM-SCC-22A, UM-SCC-38, VU-SCC-120, FaDu, VU-SCC-096, and primary oral fibroblasts. The levels of CDK1, correlated borderline significantly (two-sided *p*-value = 0.057) reverse correlation to Chk1 inhibition response (Fig. S5b). Furthermore, protein levels CDK1 were low in primary oral fibroblasts compared to the tumor cell lines. **c** Quantification of basal protein levels (Fig. 6b) of CDK1 expression levels showed a borderline significantly (two-sided *p*-value = 0.057) reverse correlation with Chk1 inhibition response (Fig. S5b). CDK1 levels might be applicable as a clinical biomarker for Chk1 inhibition response. **d** Microarray gene expression data of 22 tumors revealed a significant upregulation of *CDK1* and Cyclin B1 (*CCNB1)* in tumors when compared to the paired primary mucosa (both *p* < 0.0001, paired (two-sided) *t*-test). *CDK1* and Cyclin B1 expression varied within the patient cohort with a factor eight, but the role of stromal percentage was not taken into consideration. On the *y*-axis the relative expression level is displayed. See also legend of Fig. 1e. **e** The dose–response (*y*-axis, shown in relative cell viability) of si*CDK1* dilution range (*x*-axis) for untreated (in gray) and 750 nM LY2603618 treated (in black) conditions. Chk1 inhibition was started 24 h post transfection. Complete knockdown (mRNA < 10%, Fig. S5f) of *CDK1* resulted in resistance to Chk1 inhibition, indicating that lowering the *CDK1* levels in a high expressing cell line does not increase responsiveness to Chk1 inhibition. **f** Combination treatment of CDK4/6 inhibitor Palbociclib (EC_10_ and EC_20_) and a serial dilution of Chk1 inhibitor LY2603618/Rabusertib (*x*-axis). CDK4/6 was inhibited for 8 h (typical length of mammalian S-phase) before a serial dilution of LY2603618/Rabusertib was added. CDK4/6 inhibition partially reversed the Chk1 effects on viability. **g** The cell cycle distribution, analyzed by DNA content (PI), confirmed a partial G1/G0-arrest with the EC_10_ of Palbociclib and a total G1/G0-arrest with Palbociclib EC_20_, for both single treatment as well as in combination with LY2603618/Rabusertib. **h**, **i** Combining EC_10_ concentrations of Wee1 inhibitor Adavosertib (formerly known as AZD1775 or MK-1775) and Chk1 inhibitor LY2603618/Rabusertib, induced an additive effect in HNSCC cell lines UM-SCC-22A and VU-SCC-096. An additional EC_40_ concentration of LY2603618/Rabusertib was tested in combination with the same Adavosertib concentration as well for UM-SCC-22A. Results of combining Chk1 with Wee1 inhibition in cell line VU-SCC-120 is shown in Fig. S5j. These findings support the hypothesis that combination therapies that facilitate cell cycle progression magnify the toxicity of each of the inhibitor alone

Next, we investigated the role of DNA damage signaling and cell cycle regulation in the observed drug responses. We first analyzed DNA damage responses using histone H2Ax phosphorylation^32^ in non-transformed fibroblasts and 5 HNSCC lines (Fig. 6a). All cell lines displayed clearly increased levels of γH2Ax Ser139 after Chk1 inhibition, which was not observed in untransformed fibroblasts. Assuming that the levels of γH2Ax Ser139 accurately reflect the amount of DNA damage, this observation suggests that Chk1 inhibition triggers apoptosis in the drug-sensitive cell lines independent of the amount of DNA damage.

As reviewed Toledo et al.^33^, protein levels of CDK1 and Cyclin B1 may determine outcome of replication catastrophe (Fig. 6a, b), and could be potential predicting biomarkers. Cyclin B1 levels did not predict the response to Chk1 inhibitors in this cell line panel, but increasing levels of CDK1 did correlate with reduced sensitivity (Fig. 6b, c and S5b). The mRNA expression levels of *CDK1* and Cyclin B1 within our patient microarray database revealed a significant upregulation in HNSCC compared to the paired mucosa (Fig. 6d)^34^, with a relatively large variation of *CDK1* expression in HNSCC. This variation might reflect the relevance of CDK1 expression in HNSCC and its potential as a response biomarker.

Next, the role of CDK1 was further investigated. It has been reported that CDK1 can activate the Mek/Erk-pathway as compensatory survival mechanism of Chk1 inhibition^35^. Indeed, we noticed increased levels of p-Erk1/2 T202/Y204 in four of five cell lines after Chk1 inhibition (Fig. S5c, d), but this did not explain the difference in response.

When complexed with Cyclin A, high levels of CDK1 could also repress the effectiveness of Chk1 inhibitors by inducing late origin firing, providing a rescue mechanism for stalled S-phase. *CHEK1* depletion in mouse cells causes CDK1-Cyclin A hyper-activation and increased origin firing^36^. We questioned whether depleting *CDK1* in moderately sensitive cell lines enhanced the effect of Chk1 inhibitors, pointing to possible drug-combinations of Chk1 and CDK1 inhibitors. Since many CDK1 inhibitors also inhibit other CDKs, we tested this hypothesis by depleting *CDK1* using siRNAs, followed by addition of Chk1 inhibitor LY2603618/Rabusertib 24 h later (Fig. S5e, f). Contrary to expectations, *CDK1* knockdown was most toxic to the cells with highest CDK1 expression, suggesting an addiction to increased CDK1 levels. Moreover, to our initial surprise, *CDK1* knockdown rescued rather than aggravated the toxicity of Chk1 inhibition in all cell lines (Fig. 6e and S5e–i). We reasoned, however, that depletion of *CDK1* by siRNA might cause cell cycle arrest that precludes cells from entering S-phase, which opposes the toxic effects of Chk1 inhibition^37^. To further investigate this, UM-SCC-22A cells were co-treated with LY2603618/Rabusertib and the CDK4/6 inhibitor Palbociclib to block G1/S transition. This indeed rescued the cells, even when treated with 10-100 µM of LY2603618/Rabusertib (Fig. 6f). FACS analysis revealed that Palbociclib arrests cells in G1-phase regardless of Chk1 inhibition (Fig. 6g). Hence, lethal effects of Chk1 inhibition in HNSCC cells require S-phase entry and are (partially) reversed by G1-arrest. This is in line with the mechanism of bimodal cell killing that we presented above.

Consequently, the opposite might be true when cell cycle progression is stimulated. Wee1-like protein kinase inhibits CDK1 activity in S and G2-phases, and forms an important regulatory mechanism to halt and regulate the cell cycle^38,39^. Inhibition of Wee1 bypasses the G2/M-checkpoint and increases cell cycle progression. We therefore combined Wee1 inhibition with Chk1 inhibition, and could indeed confirm that the combination induces a more than additive effect (Fig. 6h, i).

## Discussion

Based on previous findings we investigated the canonical *ATM-CHEK2/ATR-CHEK1* pathway as specific drug target in HNSCC. *ATM, ATR*, and *CHEK2* RNA interference or drug inhibition did not comprise the viability of HNSCC cells, but *CHEK1* knockdown had major effect. For *ATM* and *ATR*, this might be due to moderate mRNA knockdown, the multiple targets of the inhibitors tested, or functional redundancy. Although sensitivities to ATR inhibitor VE-821 and Chk1 inhibitor LY2603618/Rabusertib significantly correlated, there was an absence of a therapeutic window between non-transformed cells and HNSCC cells upon ATR inhibition.

The lack of a lethal phenotype after *CHEK2* knockdown in HNSCC is more easily explained as several downstream routes are no longer intact^11,40^. In the very large majority of HNSCC, *TP53* is mutated or inactivated by the HPV protein E6^2,11,41^.

Recently, an association between *CDKN2A*/p16 deletion and sensitivity to Chk1 inhibition was postulated for HNSCC^42^. Losses of the 9p21.3 region, that contains the *CDKN2A* gene, or mutations and methylations of p16 are an early and very frequent event in squamous tumorigenesis and present in most HNSCC cell lines^2,6^. In contrast to these findings, we could not confirm any relation between p16 and Chk1 response in our cell line panel.

Intriguingly, *CHEK1* knockdown caused tumor-specific cell death of HNSCC cells in comparison to primary cells. Inhibition of Chk1 by very specific inhibitor LY2603618/Rabusertib^23,24^ demonstrated similar responses. This emphasizes dependency of HNSCC cells on functional Chk1 during DNA replication and its pivotal role to coordinate cell cycle progression in an intrinsic background of replicative stress and DNA damage^32^. All HNSCC cells displayed S-phase accumulation by Chk1 inhibition, but remarkable is the bimodal cell death mechanism: S-phase mediated apoptosis in highly sensitive cells and mitotic cell death by chromosomal breaks in moderately sensitive cell lines. The question remains whether this reflects the situation in patients, and whether mitotic cell death would contribute to response to Chk1 therapy in clinical setting. Although in vitro cell line sensitivities not always reflect the response of tumors, several successful drugs have been selected by these experiments such as gefitinib, vemurafenib and olaparib^43^.

CDK1 basal protein levels predicted response to Chk1 inhibition^33^. CDK1 regulates many cell cycle phases, with a critical role in mitotic entry^39^. The activity of CDK1 during G2/M-transition is blocked by Wee1 through Y15 phosphorylation, while CDK1 can be activated by CDK-activating phosphatase CDC25C^39^. During the initiation of the M-phase, CDC25C removes the CDK1 phosphate Y15, whereupon activated Cyclin B-CDK1 inhibits Wee1 activity through phosphorylation in a feed forward loop. Simultaneously, active CDK1 enforces Chk1 translocation from the nucleus to the cytoplasm through hyper-phosphorylation^38,44^. Partial phosphorylating activation or “priming” of Chk1 by CDK1 results in a cell cycle arrest, thereby regulating timing of mitotic entry^44,45^. Besides the G2/M-checkpoint, CDK1 plays an important role in other cell cycle phases, among which the activation of MAPK/Erk-pathway and the regulation of late origin firing in S-phase when complexed with Cyclin A^35–37,39,46,47^. To execute late S-phase activities, low levels of CDK1 activation are demanded, while mitotic entry requires high levels of CDK1 activity^39^.

Complete knockdown of CDK1 results in abrogation of S-phase control, followed by delayed cell cycle progression and G2-arrest^48^. Others showed that CDK1 inhibitor RO-3306 not always initiates a full G2-arrest^49^. In vitro, the combination of *CDK1* knockdown with Chk1 inhibition reversed the Chk1 effect in HNSCC, and similar effects were observed using the CDK4/6 inhibitor Palbociclib. CDK4/6 are conserved regulators of the G1-phase and complexing with Cyclin D is essential for G1-to-S-phase progression^50^. Palbociclib was recently approved for breast cancer^51^.

**Fig. 7:**
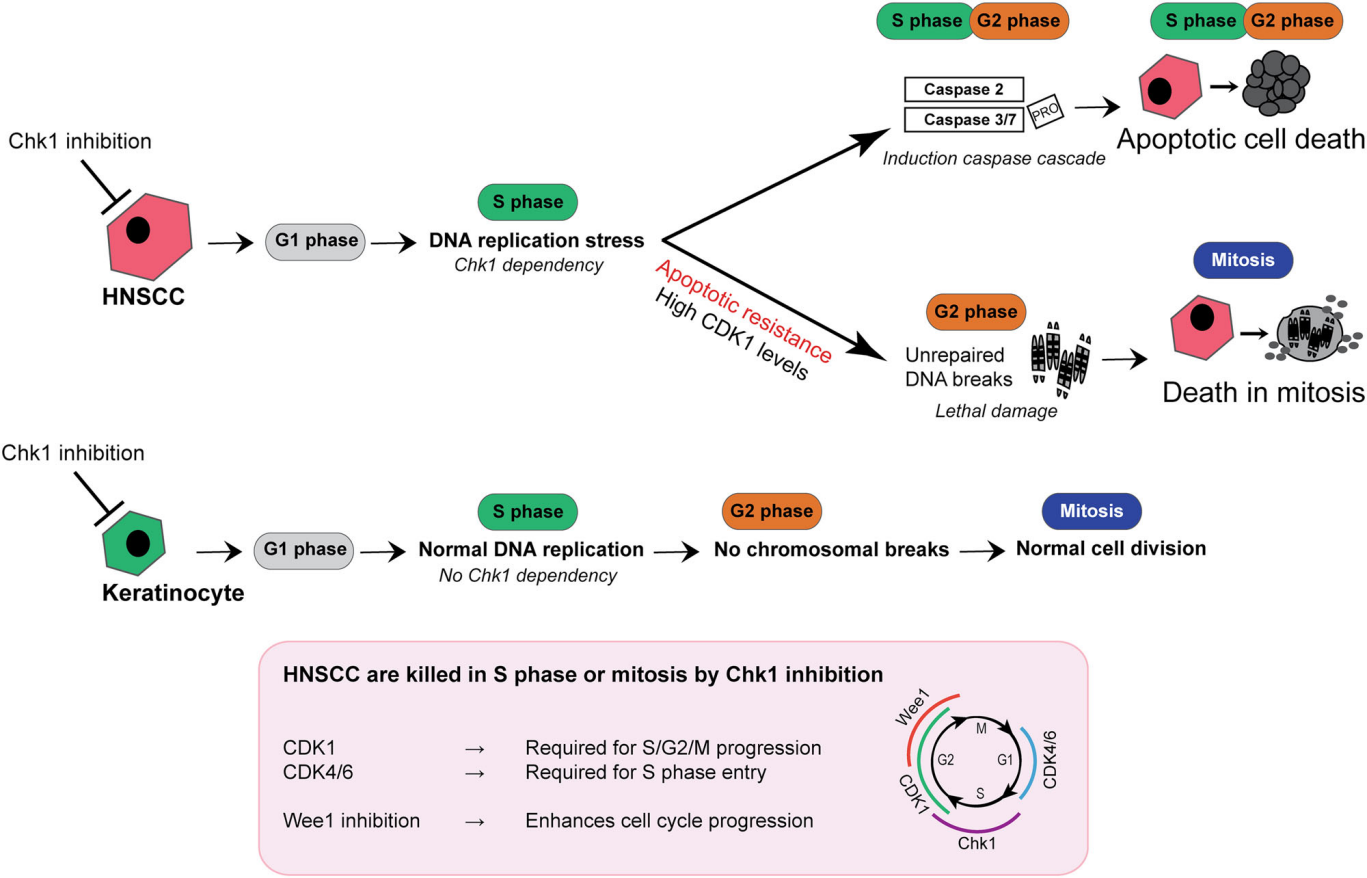
Schematic summary of molecular mechanism. Schematic summary of molecular mechanism underlying Chk1 sensitivity in HNSCC. When Chk1 is inhibited, cells accumulate in S-phase due to DNA replication problems. This results in increased DNA damage (γH2Ax Ser139). Sensitive cells initiate a caspase cascade in S- or early G2-phase, to initiate apoptotic cell death. These cells typically harbor a low intrinsic CDK1 level. Less-sensitive cells encounter comparable DNA replication problems due to Chk1-dependency, but do not initiate apoptosis and proceed into mitosis with incomplete DNA replication and unrepaired DNA damage. This results in chromosomal breaks causing death in mitosis during subsequent cell division. Primary cells lack the strict Chk1-dependency during DNA replication, due to a tightly regulated cell cycle control, therefore, primary cells enable to successfully continue into mitosis when Chk1 is inhibited. Our data further shows that the efficacy of Chk1 inhibition in HNSCC is dependent on cell cycle progression. Therefore, functional CDK4/6 is required for S-phase entry and functional CDK1 is necessary for S-phase progression and subsequent mitotic entry, thereby facilitating the accumulation of DNA damage. The combination of Chk1 inhibitors with therapies that hamper cell cycle progression should not be considered for clinical research, but combination with inhibitors that support cell cycle progression seems to have more than added value

In summary, our data indicate that loss of Chk1 activity leads to severe DNA replication problems in HNSCC (Fig. 7), collapsed replication forks and subsequent S-phase accumulation and DNA damage. In relation to CDK1 expression levels, a bimodal response is observed. Cells with low CDK1 levels are very sensitive and undergo S-phase replication catastrophe by caspase-mediated apoptosis. Cells with high CDK1 levels are CDK1-addicted, but are less sensitive to Chk1 inhibition, resist S-phase apoptosis, but nonetheless die in mitosis by chromosomal breaks. Chk1 inhibition should not be combined with CDK1 or CDK4/6 inhibitors, or other drugs that hamper cell cycle progression, as cell cycle progression is essential for effective Chk1 inhibition. Combination with inhibitors that stimulate cell cycle progression have an additive effect.

Dual Chk1/Chk2 inhibition with LY2606368/Prexasertib is less HNSCC specific, likely due to the dual targeting of this inhibitor, and which may relate to observed toxicities^52^. Our data indicate that particularly specific Chk1 inhibitors should be considered for clinical applications in HNSCC. In the first published phase I/II clinical trials, combination therapies were applied with LY2603618/Rabusertib and either Cisplatin and Pemetrexed, or Pemetrexed alone, or Gemcitabine. Despite the combination, acceptable safety was reported in 6 out of 7 studies, and partial responses, stable disease and increased overall survival was achieved in these initial studies^53–59^. Based on our study and data of others^60^, a phase I/II clinical trial with a highly specific Chk1 inhibitor with or without cell cycle enhancing therapy such as Wee1 inhibition may be initiated for recurrent/metastatic HNSCC. Basal CDK1 expression should be used as a potential biomarker for response to Chk1 inhibition or even as a selection criterion for enrollment.

## Materials and methods

### Lethality scores, siRNA transfections, and viability assays

Lethality scores of independent siRNA screens was established as published^20^. Cell culture conditions, siRNA transfections, and viability assays using CellTiter-Blue® (Promega, Leiden, The Netherlands) as described previously^19,21,61^. Primary oral fibroblasts and keratinocytes were obtained from resected uvulas from healthy individuals undergoing uvulopalatopharyngoplasty, according to the Dutch Medical Scientific Societies guidelines and the Dutch regulations on medical research^61^. Cell lines were authenticated regularly, using *TP53* mutations, examination for high-risk human papillomavirus (HPV) by PCR^22^, other genetic markers and morphological characteristics. Cell lines were regularly tested for mycoplasma (Mycoalert, Lonza, Verviers, Belgium).

### Quantitative reverse transcription PCR (RT-qPCR)

RNA was isolated with PureLink RNA micro kit (Thermo Fisher, Bleiswijk, The Netherlands), RNA was synthesized into cDNA using Taqman® Reverse Transcription Reagents (Life technologies, Bleiswijk, The Netherlands) with random primers, and gene expression was analyzed using power SYBR® Green PCR Master Mix (Thermo Fisher) 24 h post-transfection in triplicate. Primers sequences of *ATM*, *ATR*, *CHEK1*, *CHEK2* and *GUSB* (housekeeping gene) were obtained from the qPrimer Depot^62^ and obtained from BioLegio (Nijmegen, the Netherlands). Probes for *CDKN2A* (Hs00939627_m1), *CDK1* (Hs00938777_m1) and *GUSB* (Hs00355782_m1) were used in the Applied Biosystems Taqman gene expression assay (Life technologies).

### Expression microarray

Database GEO accession number GSE83519^21,34,63^.

### Low coverage whole-genome sequencing for 9p21.3

Genomic DNA was isolated, sheared, and a library prepared and sequenced by low coverage whole-genome sequencing as described^64^. Sequencing was performed on a HiSeq 2500 (Illumina, Eindhoven, The Netherlands) using 150 bp single ended runs.

### Dose–response curves with small molecule inhibitors

Short-term (72 h drug exposure) was performed as previously described^21^.

For long-term exposure, cells were seeded at low density in a six-well plate. Treatment started 24 h later, and refreshed twice weekly until 80% confluency. Cells were stained using crystal violet (Sigma-Aldrich) after fixation using 2% paraformaldehyde (Sigma-Aldrich, Zwijndrecht, The Netherlands).

KU-60019, Wortmannin, ETP-46464, VE-821, MK-8776 (SCH 900776), PF-477736, LY2603618/Rabusertib, and Palbociclib were purchased from Selleckchem (Munich, Germany), LY2606368/Prexasertib from Medchem (Sollentuna, Sweden). Adavosertib (MK-1775) from Biovision (Milpitas, USA). All were dissolved in a DMSO stock dilution (10 mM), except Palbociclib (10 mM in ddH_2_O). Assays contained <1% DMSO. All drug-survival assays depict the standard error of the mean (SEM) of three independent experiments in triplicate.

### Flow cytometry

DNA content was measured per cell with propidium iodide (5 µg PI per 10^6^ cells, Sigma-Aldrich) staining, gating 20,000 events^21^. Replication and DNA content was obtained using BrdU/PI. Cells were pulsed with 4 nmol/L 5-bromo-20-deoxyuridine (BrdU, Sigma-Aldrich) for 15 min. After trypsinization and correcting for input number, cells were fixed in 75% ethanol overnight. After 0.5 mg/mL RNAse A incubation, cells were permeabilized with 5 mol/L HCl:0.5% Triton X-100 for 20 min at RT thereafter neutralized with 0.1 mol/L Na_2_B_4_O_7_. Mouse-anti-BrdU (clone Bu20a) antibody (M0744, Agilent technologies, Amstelveen, The Netherlands) was incubated overnight at 4 °C, followed by a fluorescein isothiocyanate (FITC)-conjugated rabbit anti-mouse antibody (F0313, Agilent technologies) and propidium iodide (5 µg PI per 10^6^ cells, Sigma-Aldrich). BD LSR II Fortessa™ (BD Biosciences, Vianen, The Netherlands) and BD FACSDiva™ software (V8.0.1, BD Biosciences) were used for flow cytometry and data analysis.

### Time-lapse microscopy

Cells were seeded on a 35 mm glass-bottom dish (Willcowells, Amsterdam, The Netherlands) 48 h prior imaging as previously described^65^. Images were analyzed using MetaMorph software (Universal Imaging, Bedford Hills, USA) and ImageJ Fiji *Metamorph nd & ROI files importer (nd stack builder)* plugin^66^.

### Western blot analysis

Normalized whole cell lysates were run on 4–12% pre-casted gradient sodium dodecyl sulfate polyacrylamide gel electrophoresis gels (Bolt Bis-Tris Plus gels, Thermo Fisher) and developed using Amersham Hyperfilm™ ECL (GE Healthcare) or Uvitec 47 Alliance reader (Uvitec Cambridge, UK). All antibodies are listed in Table S4.

### Apoptosis, necrosis, and viability assay

Relative apotosis, necrosis, and viability was determined using the ApoTox-Glo™ Triplex Assay (Promega), according to manufacturer’s protocol in triplicate.

### Metaphase analysis

Cells were seeded at day 0 in a T75 flask. After 72 h recovery, cells were treated with LY2603618/Rabusertib for 24 h. Subsequently, protocol was followed as described previously^67^.

### Statistical analysis

All statistics were performed in GraphPad Prism version 8, and R version 3.4.3. The heatmap was obtained using the *heatmap.2 function* of the R package *gplots*. All figures represent at least triplicate median values, experiments were repeated multiple times and representable experiments and SD are shown.

## Supplementary information

Supplementary file

Supplementary figure S1

Supplementary figure S2a-b

Supplementary figure S2c-d

Supplementary figure S2e-g

Supplementary figure S3

Supplementary figure S4

Supplementary figure S5
