# Supplementary material for: Targeting the cell cycle in head and neck cancer by Chk1 inhibition: a novel concept of bimodal cell death
Source: Oncogenesis. 2019 Jun 17;8(7):38. doi: 10.1038/s41389-019-0147-x (PMC6572811; doi:10.1038/s41389-019-0147-x)
Supplement: Supplementary file 1 — Supplementary file [file 41389_2019_147_MOESM1_ESM.pdf]

Supplementary data

Targeting the cell cycle in head and neck cancer by Chk1 inhibition: a novel concept of bimodal cell death

Anne M. van Harten[1], Marijke Buijze[1], Richard van der Mast[1], Martin A. Rooimans[2], Sanne R. Martens-de Kemp[1], Costa Bachas[1], Arjen Brink[1], Marijke Stigter-van Walsum[1], Rob M. F. Wolthuis[2], Ruud H. Brakenhoff[1]

[1] Department of Otolaryngology/Head and Neck Surgery, section Tumor Biology, Amsterdam UMC, VUmc Cancer Center Amsterdam, The Netherlands

[2] Department of Clinical Genetics, section Oncogenetics, Amsterdam UMC, VUmc Cancer Center Amsterdam, The Netherlands

Corresponding author:

Ruud H. Brakenhoff, Amsterdam UMC, VUmc Cancer Center Amsterdam, Department of Otolaryngology/Head and Neck Surgery, PO Box 7057, 1007 MB Amsterdam, The Netherlands. rh.brakenhoff@vumc.nl. Phone: +31-20-4440953. Fax: +31-20-4443688.

Supplementary number of references: 2
Supplementary tables: 4
Supplementary figures + legends: 5

Supplementary figure S1. RNA interference of *CHEK1* decreases cell viability in HNSCC cell lines, but not in primary oral keratinocytes and fibroblasts.

a.
Lethality scores per gene of all individual replicates from the genome-wide siRNA screens, performed in HNSCC cell lines VU-SCC-1131 and VU-SCC-120 (Figure 1b). Values are represented in boxplots, with the horizontal line representing the median lethality score. Two-sided t-testing showed significant differences in lethality for *CHEK1* knockdown compared to *ATM*, *ATR* and *CHEK2*.

b.
Deconvolution of the 4 individual SMARTpool siRNAs on 5 additional HNSCC cell lines in addition to Figure 1f. Both HPV-negative (VU-SCC-OE, FaDu and VU-SCC-096, in red) and HPV-positive (VU-SCC-147 and UM-SCC-47, in yellow) cell lines were tested. A significant decreased viability was observed in all tested HNSCC cell lines (two-sided t-test p values siCONTROL#2 vs si*CHEK1* pool: VU-SCC-OE <0.0001, UM-SCC-47 <0.0001, VU-SCC-147 0.0007, FaDu 0.0004, VU-SCC-096 <0.0001). siCONTROL#2 was transfected as negative control, si*UBB* (targeting Ubiquitin B) as positive transfection control.

c.
Analysis of mRNA knockdown by RT-qPCR of all individual siRNAs targeting *CHEK1* in cell line UM-SCC-47 was isolated 24h post-transfection.

Supplementary figure S2. Dose-responses to treatment with small molecule inhibitors targeting ATM, ATR and Chk1.

Small molecule inhibitors targeting ATM (KU-60019 and Wortmannin), ATR (ETP-46464 and VE-821), and Chk1 (MK8776, PF-477736 and LY2603618/Rabusertib) were tested on a panel of HPV-negative (UM-SCC-22A, UM-SCC-38, VU-SCC-OE and VU-SCC-120) as well as HPV-positive (UM-SCC-47 and VU-SCC-147) HNSCC cell lines (depicted in red), and were compared to the responses of primary oral keratinocytes and fibroblasts (in short P.O. fibro./kera. 1 donor each, depicted in green). Experiments were performed three times in triplicate and the averaged value is indicated. Cell viability is shown after 72h drug treatment.

a.
Responses for ATM inhibitor KU-60019 were equal for HNSCC cell lines and primary squamous cells.

b.
ATM inhibitor Wortmannin did not induce tumor cell-specific viability reduction in tested HNSCC cell lines.

c.
ETP-46464, an inhibitor of both mTOR and ATR reduced cell viability similarly in HNSCC cell lines as in primary oral fibroblasts and keratinocytes.

d.
ATR inhibitor VE-821 did cause a small reduction in cell viability in UM-SCC-22A and VU-SCC-OE compared to primary controls, but not for other tested cell lines.

e.
Chk1 inhibitor MK-8776 showed an increase of cell viability after initial effect on cell viability, in all HPV-negative cell lines with concentrations of 1 μM and higher. This remarkable effect is likely caused by off-target activity of this small molecule inhibitor, given all the other data in this manuscript.

f.
Low concentrations of Chk1 inhibitor PF-477736 caused a decreased in the viability of primary squamous cells, which was not in concordance with the observed effects of RNA interference.

g.
An additional panel of HNSCC cell lines tested with LY2603618/Rabusertib (Figure 2a).

Supplementary figure S3. Chk1, γH2Ax Ser139 and p16 intrinsic protein levels do not correlate with EC50 responses to Chk1 inhibition using LY2603618/Rabusertib. Cell cycle distribution upon Chk1 inhibition.

a.
Basal proteins levels of Chk1 and γH2Ax Ser139 of HNSCC cell lines UM-SCC-22A, UM-SCC-38, VU-SCC-120, FaDu, VU-SCC-096 and primary oral fibroblasts. HSP90α/β was used as loading control.

b.
Pearson correlation of Chk1 basal protein levels and the EC_{50} of LY2603618/Rabusertib. A near significant correlation was found ($p=0.15$, $R=0.74$, $\alpha=0.05$).

c.
Pearson correlation of DNA damage (γH2Ax Ser139 phosphorylation) and the EC_{50} of LY2603618/Rabusertib. No correlation was found ($p=0.47$, $R=0.433$, $\alpha=0.05$).

d.
mRNA expression analyzed by RT-qPCR of *CDKN2A*/p16 is shown, corrected for cDNA input by ΔCT levels of housekeeping gene *GUSB*. Cell lines depicted with a value of -10 or lower did not have a ΔCT value, corresponding with absence of *CDKN2A*/p16 expression.

e.
Western blot analysis of p16. The panel of 5 HPV-negative cell lines, ordered to Chk1 inhibition sensitivity, did not contain any detectable p16 protein, where 2 HPV-positive control cell lines did show p16. The upper band might correspond with p21 as previously suggested by Yan Li *et al.*[1]

f.
Cell cycle distribution based on DNA content after 24h of LY2603618/Rabusertib treatment is shown. Extended panel of cell lines from figure 3a. All cell lines showed an increased S-phase population after treatment. Relative DNA content is depicted.

g.
UM-SCC-22A was treated with 750 nM LY2603618/Rabusertib. For 24h, every 2h cells were harvested to analyze the cell cycle distribution by PI bound DNA content with flow cytometry. The S-phase fraction remained equal for the first 8h of Chk1 inhibition. After 8h of treatment, the population of S-phase cells was increasing, which suggests DNA replication problems in early S-phase and subsequent accumulation of S-phase cells.

h.
Cell cycle distribution (DNA content) after 24h of LY2606368/Prexasertib is shown. Relative DNA content is depicted. An S-phase increase was found for all cell lines after 24h treatment, except VU-SCC-096.

Supplementary figure S4. Time lapse microscopy revealed different mechanisms of cell death between sensitive and moderately sensitive HNSCC cell lines, which was confirmed by caspase 3/7 and necrosis assay.

a.
Mitotic lengths of sensitive cell line UM-SCC-22A are shown. Without treatment (left panel), mitotic length of unsuccessful divisions was not significantly elongated (two-sided t-test p=0.3). During Chk1 inhibition (middle panel), unsuccessful mitosis was significantly elongated (two-sided t-test p=0.0002). Successful mitotic length was not significantly different (right panel) between untreated and treated cells (two-sided t-test p=0.77), but the length of unsuccessful mitosis significantly differed between treated and untreated cells, potentially due to the amount of DNA damage and pre-mature mitotic entry of treated cells (two-sided t-test p=0.038).

b.
Mitotic length was measured from the time laps footage. For moderately sensitive cell line VU-SCC-096, a significantly difference in mitotic length was found without treatment (left panel) (two-sided t-test p<0.001). During Chk1 inhibition (middle panel), the mitotic length between successful mitosis and cells that undergo mitotic cell death remains significantly different (two-sided t-test p=0.013). However, mitotic length between treated and untreated cells (right panel) does not differ, nonetheless the mitosis is successful (two-sided t-test p=0.46) or ends in mitotic cell death (two-sided t-test p=0.97).

c.
Apoptosis, necrosis and viability was accessed 24h post-treatment in cell lines UM-SCC-22A, VU-SCC-120 and VU-SCC-096 using a serial dilution of LY2603618/Rabusertib. In UM-SCC-22A cells caspase 3/7 mediated apoptosis was activated, whereas in VU-SCC-120 both apoptosis and necrosis was induced. VU-SCC-096 mainly showed cytotoxicity (necrosis) after 24h with increasing concentrations of LY2603618/Rabusertib.

Supplementary figure S5. Molecular pathway analysis of cell cycle regulators.

a.
Western blot analysis of UM-SCC-22A cells treated with LY2603618/Rabusertib showed over time an increase of DNA damage markers p-ATM Ser1981 and γH2Ax Ser139. Mitotic entry marker p-H3 Ser10 decreased, in line with cell cycle distributions obtained by DNA content analysis.

b.
Pearson correlation of basal CDK1 levels and EC_{50} of LY2603618/Rabusertib. A borderline correlation was found for cell lines UM-SCC-22A, UM-SCC-38, VU-SCC-120, FaDu and VU-SCC-096 ($R=0.87$, $p(\text{two-tailed})=0.06$, $\alpha=0.05$).

c.
Western blot analysis depicting MAPK/Erk pathway activation upon Chk1 inhibition. p-Erk1/2 T202 / Y204 is found upregulated in all cell lines except FaDu after 24h treatment.

d.
MAPK/Erk pathway activation in most sensitive cell line UM-SCC-22A and moderately responding cell line VU-SCC-096 is time dependent. UM-SCC-22A rapidly up-regulates p-Erk1/2 T202/Y204 after 4h, VU-SCC-096 full activation is seen after 12h of exposure.

e.
Knockdown of *CDK1* did not decrease cell viability in UM-SCC-22A (98% relative viability to siCONTROL#2), but reduced the cell viability in VU-SCC-096 to 68%. This might indicate different roles of *CDK1* in these cell lines.

f.
CDK1 mRNA knockdown 24h post-transfection with si*CDK1*. <10% mRNA of *CDK1* remained in VU-SCC-096.

g. – i.
CDK1 knockdown reverses the effect on viability of the Chk1 inhibitor in VU-SCC-096, FaDu and UM-SCC-22A. Chk1 inhibition started 24h after siRNA transfection with siRNA SMARTpool *CDK1*.

j.
(Extension of figure 6h-i). Combining EC_{10} concentrations of Wee1 inhibitor Adavosertib (formerly known as AZD1775 or MK-1775) and Chk1 inhibitor LY2603618/Rabusertib, also induced an additive effect in HNSCC cell line VU-SCC-120.

Supplementary table S1: Live cell imaging

**Cell line**		***# of cells***	**Cell fate**
**UM-SCC-22A**	Untreated	3/50	Death in mitosis
	750 nM LY2603618/Rabusertib	6/50	Death in mitosis
		33/50	Apoptotic cell death
**VU-SCC-096**	Untreated	3/50	Death in mitosis
	750 nM LY2603618/Rabusertib	27/50	Death in mitosis
		10/50	Apoptotic cell death

Supplementary table S2: Dharmacon siRNA sequence

Target		siRNA sequence
ATM	siRNA SMARTpool sequences	*GCAAAGCCCUAGUAACAUA*
		GGGCAUUACGGGUGUUGAA
		UCGCUUAGCAGGAGGUGUA
		UGAUGAAGAGAGACGGAAU
ATR	siRNA SMARTpool sequences	*GAACAACACUGCUGGUUUG*
		GCAACUCGCCUAACAGAUA
		UCUCAGAAGUCAACCGAUU
		GAAUUGUGUUGCAGAGCUU
CHEK1[§]	siRNA sequence #6	*GCAACAGUAUUUCGGUAUA*
	siRNA sequence #7	*GGACUUCUCUCCAGUAAAC*
	siRNA sequence #8	*AAAGAUAGAUGGUACAACA*
	siRNA sequence #26	*AGAUAUGAAGCGUGCCGUA*
CHEK2	siRNA SMARTpool sequences	*GAAAUUGCACUGUCACUAA*
		CUCAGGAACUCUAUUCUAU
		AAACGCCGUCCUUUGAAUA
		GCUAAAUCAUCCUUGCAUC
CDK1	siRNA SMARTpool sequences	*GUACAGAUCUCCAGAAGUA*
		GAUCAACUCUUCAGGAUUU
		GGUUAUAUCUCAUCUUUGA
		GAACUUCGUCAUCCAAAUA

§ *The SMARTpool consisted of these 4 individual siRNA sequences.*

Supplementary table S3: RT-qPCR SYBR green primer sequences

Target		Primer sequences§
ATM	Forward	*TTCAAAGGATTCATGGTCCAG*
	Reverse	*GCTGTGAGAAAACCATGGAA*
ATR	Forward	*AACATTCGTGGCATTGACTG*
	Reverse	*AAGCAAGGTGATCTCATCCG*
CHEK1	Forward	*TCATCCATTTCTAACAAATTCACTT*
	Reverse	*TGGGCTATCAATGGAAGAAAA*
CHEK2	Forward	*TCGAAAGCCAGCTTTACCTC*
	Reverse	*TGATCAGTCAGTTTATCCTAAGGC*
GUSB	Forward	*GAAAATATGTGGTTGGAGAGCTCATT*
	Reverse	*CCGAGTGAAGATCCCCTTTTTA*

§ *QRT-PCR primer sequences were obtained from the qPrimer Depot.*[2]

Supplementary Table S4: Western blot antibodies

Antibody target	Phospho-site	Clone	Cat. Nr.	Company
**ATM**		2C1	sc23921	Santa Cruz, Heidelberg, Germany
**p-ATM**	Ser1981	10H11.E12	sc47739	Santa Cruz, Heidelberg, Germany
**β-actin**		N-21	sc130656	Santa Cruz, Heidelberg, Germany
**Caspase-2**			AF826	R&D systems, Abingdon, UK
**CDK1**			AB47779	Abcam, Cambridge, UK
**Chk1**			sc8408	Santa Cruz, Heidelberg, Germany
**Cyclin B1**		V152	#4135S	Cell Signaling, Bioké, Leiden, The Netherlands
**p44/p42 MAPK (ERK1/2)**		137F5	#4695	Cell Signaling, Bioké, Leiden, The Netherlands
**p-p44/p42 MAPK (p-ERK1/2)**	T202/Y204		#9101S	Cell Signaling, Bioké, Leiden, The Netherlands
**γH2Ax**	Ser139		AF2288	R&D systems, Abingdon, UK
**HSP90α/β**		F-8	sc13119	Santa Cruz, Heidelberg, Germany
**p16**			554079	BD Pharmingen, Vianen, The Netherlands

SUPPLEMENTARY REFERENCES

1 Li Y, Nichols MA, Shay JW, Xiong Y. Transcriptional repression of the D-type cyclin-dependent kinase inhibitor p16 by the retinoblastoma susceptibility gene product pRb. *Cancer Res* 1994; **54**: 6078–6082.

2 Cui W, Taub DD, Gardner K. qPrimerDepot: a primer database for quantitative real time PCR. *Nucleic Acids Res* 2007; **35**: D805-9.
