# Supplementary figures and images for: Targeting the cell cycle in head and neck cancer by Chk1 inhibition: a novel concept of bimodal cell death
Source: Oncogenesis. 2019 Jun 17;8(7):38. doi: 10.1038/s41389-019-0147-x (PMC6572811; doi:10.1038/s41389-019-0147-x)

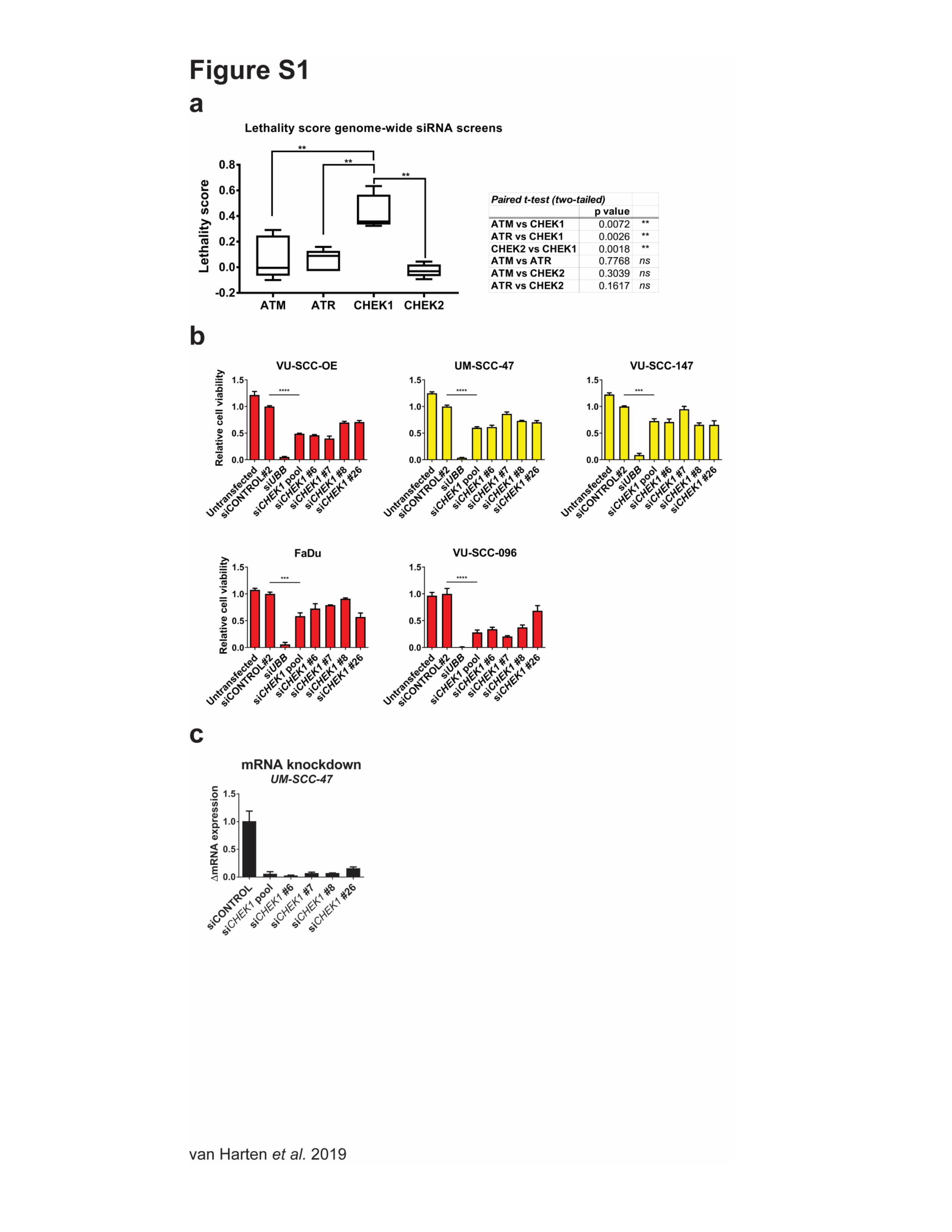

Supplement: Supplementary file 2 — Supplementary figure S1 [file 41389_2019_147_MOESM2_ESM.tif]

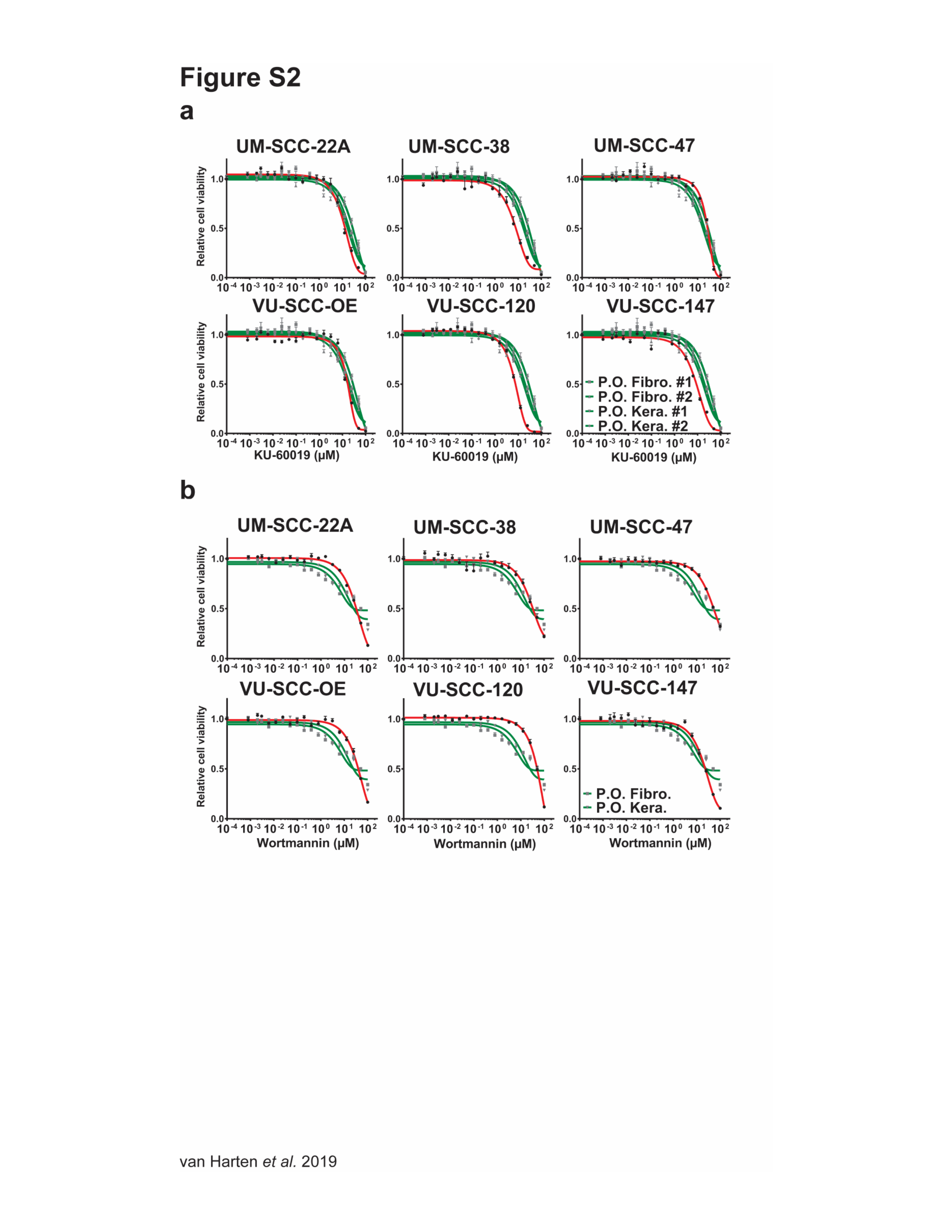

Supplement: Supplementary file 3 — Supplementary figure S2a-b [file 41389_2019_147_MOESM3_ESM.tif]

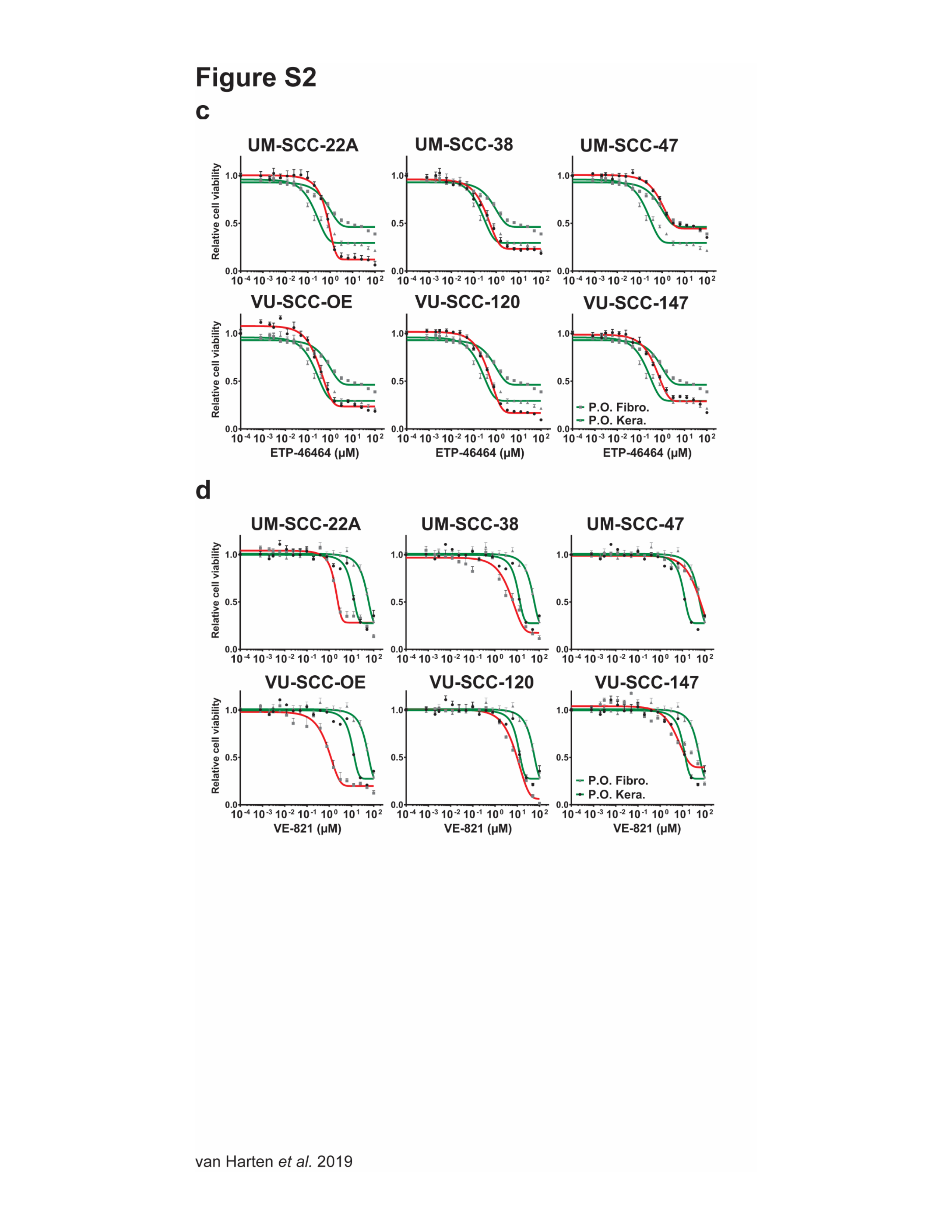

Supplement: Supplementary file 4 — Supplementary figure S2c-d [file 41389_2019_147_MOESM4_ESM.tif]

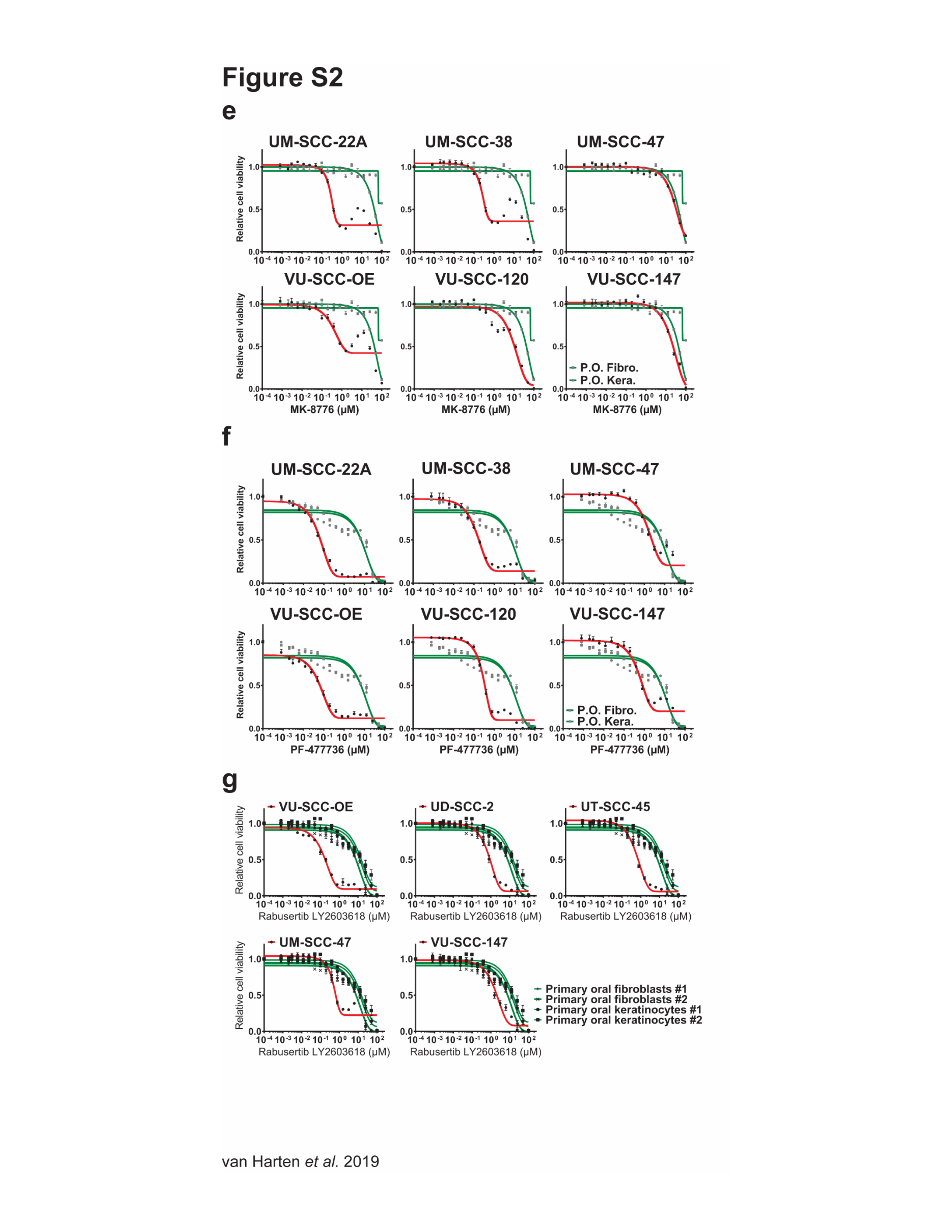

Supplement: Supplementary file 5 — Supplementary figure S2e-g [file 41389_2019_147_MOESM5_ESM.tif]

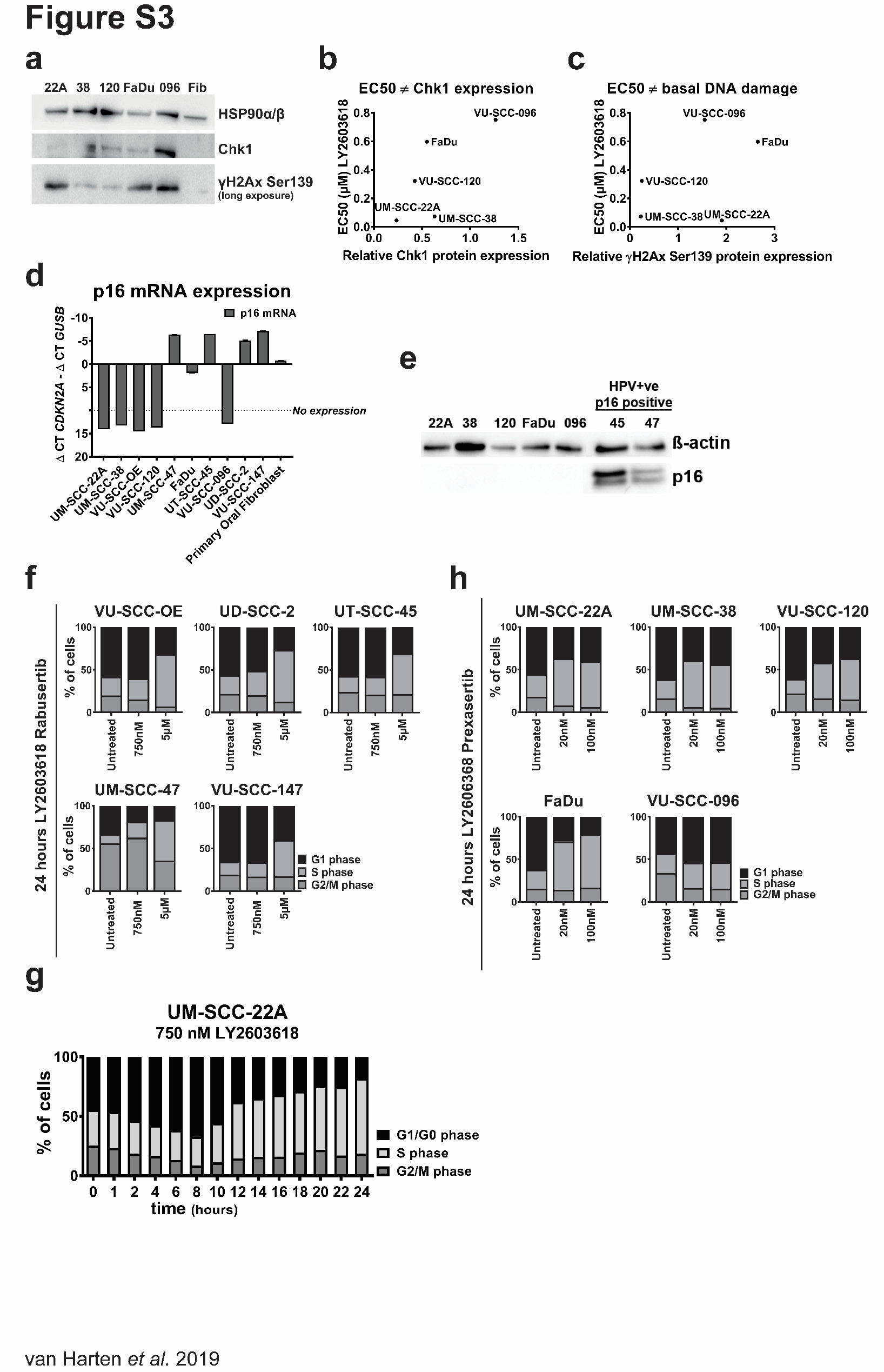

Supplement: Supplementary file 6 — Supplementary figure S3 [file 41389_2019_147_MOESM6_ESM.tif]

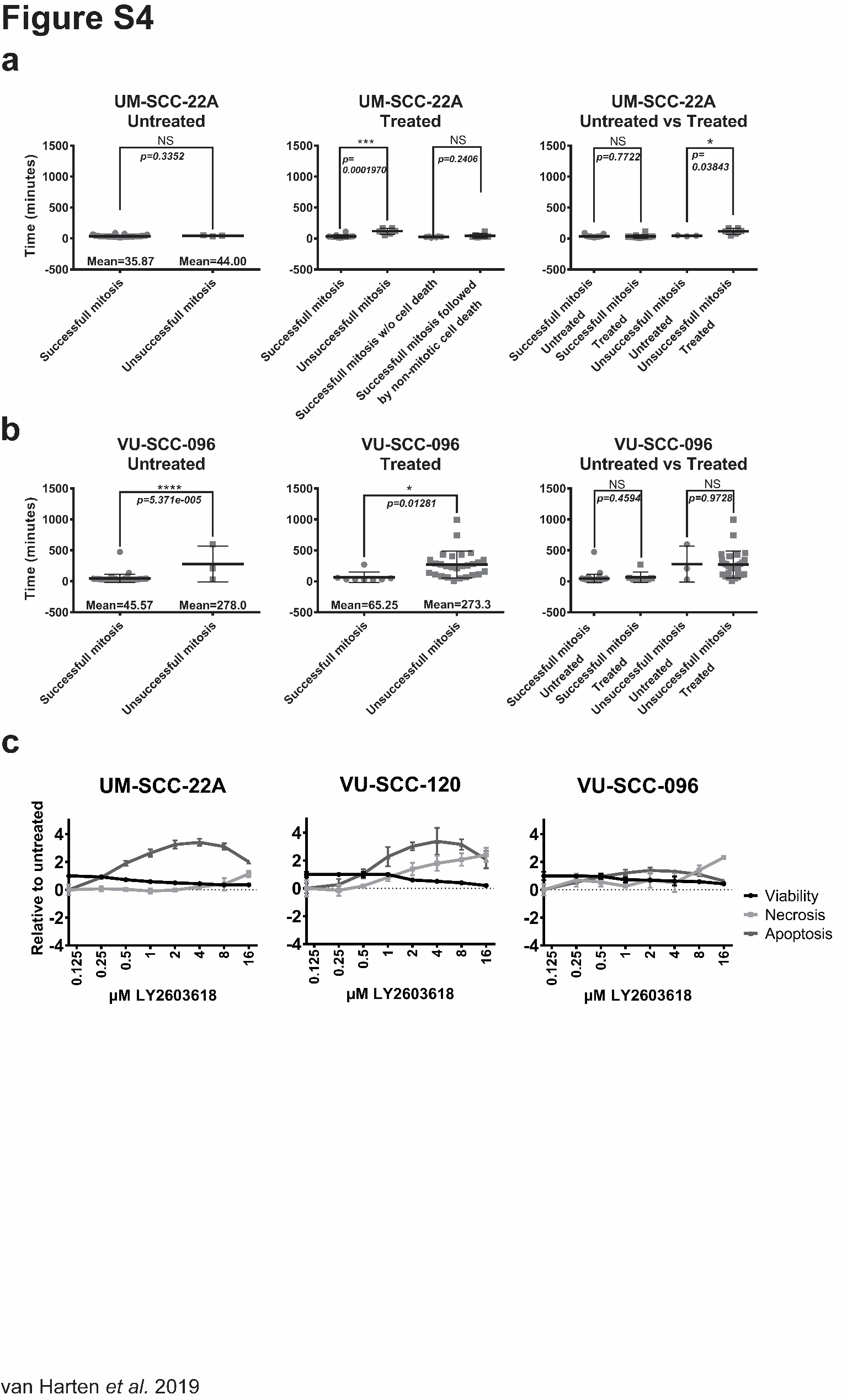

Supplement: Supplementary file 7 — Supplementary figure S4 [file 41389_2019_147_MOESM7_ESM.tif]

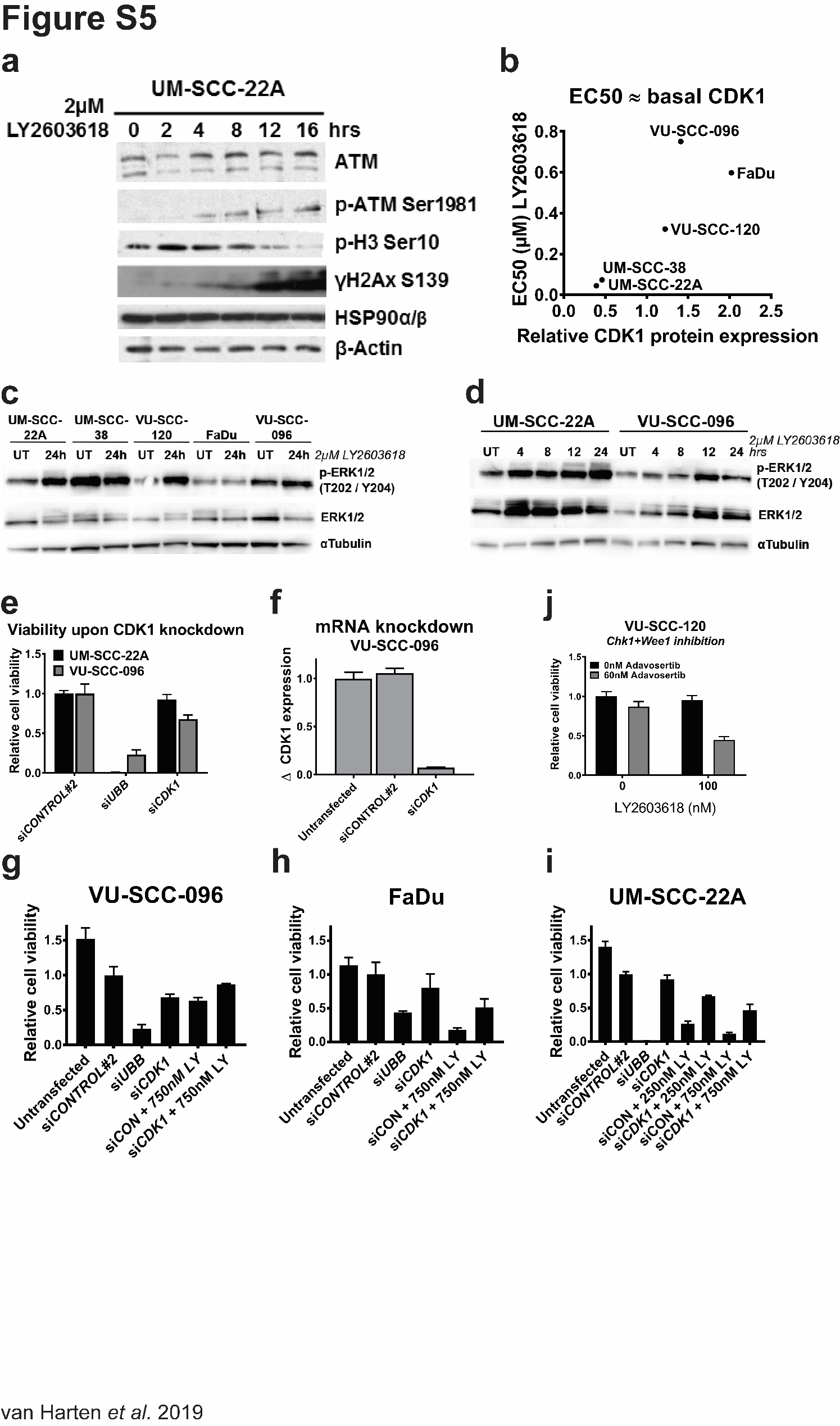

Supplement: Supplementary file 8 — Supplementary figure S5 [file 41389_2019_147_MOESM8_ESM.tif]
